# Three cluster randomised controlled trials in Portugal, Czechia and the United Kingdom to test the effectiveness and cost-effectiveness of an augmented social play intervention in schools to support real-world connection and sense of belonging (*Lina* trial protocol)

**DOI:** 10.1186/s13063-026-09780-7

**Published:** 2026-06-03

**Authors:** Tamanna Malhotra, Onaiza Qureshi, Adam Barnard, Victoria Jane Bird, Patrícia Brás, Vítor Alexandre Coelho, Alison Fulop, Yan Feng, Tomáš Hunčík, Marta Marchante, Philip McNamee, Gloria Mittmann, Nyareso Mokaya, Adéla Petřeková, Ana Cláudia Primor, Čeněk Šašinka, Alžběta Šašinková, Beth Stuart, Kate Anne Woodcock

**Affiliations:** 1 School of Psychology, University of Birmingham, Birmingham, UK; 2 Wolfson Institute of Population Health, Queen Mary University of London, London, UK; 3 Adam Barnard and Associates, Brighton, UK; 4 Public Health and Wellbeing, University of Essex, Colchester, UK; 5 Académico de Torres Vedras, Torres Vedras, Portugal; 6 Masaryk University, Brno, Czech Republic; 7 Research Centre Transitional Psychiatry, Karl Landsteiner University of Health Sciences, Krems an Der Donau, Austria; 8 Institute of Mental Health and Centre for Developmental Science, School of Psychology, University of Birmingham, Birmingham, UK

**Keywords:** Adolescent mental health, Classroom belonging, Loneliness, Augmented social play, School-based intervention, Cluster randomised controlled trial, Digital health, E-health

## Abstract

**Background:**

Mental ill-health and loneliness represent major challenges for today’s youth, exacerbated by unprecedented societal demands, including concurrent socialisation in physical and digital worlds. A strong sense of belonging can buffer against these challenges, and in adolescents is largely constituted in schools. Augmented Social Play (ASP) involves smartphone-led cooperative face-to-face gameplay which integrates immersive storytelling, augmented reality, and evidence-based psychology to strengthen belonging. Building on this foundation, *Lina* is a multi-session intervention designed for classroom-based delivery during routine school lessons.

**Methods:**

Three parallel hybrid type II cluster pragmatic randomised controlled trials will be conducted in secondary schools in Czechia, Portugal and the UK. The independently powered and analysed trials will examine the effectiveness of *Lina*—compared to standard lessons—in strengthening classroom belonging, reducing symptoms of mental ill-health and improving wellbeing, loneliness and stigma. Forty classes of students whose modal age is 12–13 years will participate in each country. Effectiveness will be examined on completion of *Lina* and after 6 months, using the student-report Delaware School Climate Survey, the Social Anxiety Scale for Adolescents, the Patient Health Questionnaire-8, the Social Awareness subscale of the Social-Emotional Learning Edition of the Strengths and Difficulties Inventory System, the WHO-5 Well-being Index, the UCLA Loneliness Scale and a tailored version of the Peer Mental Health Stigmatisation Scale-Revised. To examine cost-effectiveness, quality of life will be measured in a corresponding manner, using the Child Health Utility 9D and the EQ-5D-Y-3L, and analysed alongside bespoke resource-use inventories, completed via teachers and/or school records. An embedded developmental evaluation of implementation will draw on researcher observations of *Lina*, teacher-reported Feasibility, Acceptability and Appropriateness of Intervention questionnaires and teacher focus groups. A second developmental phase of implementation evaluation will follow the effectiveness trial, while control classes do *Lina* with their teachers. An integrated qualitative evaluation will examine students’ experiences of *Lina* using individual interviews and focus groups, including a particular focus on students with characteristics that put their sense of belonging at risk.

**Discussion:**

Findings will speak to the potential impact of Lina on youth mental health. They will also inform Lina implementation guidance for diverse national and international school contexts.

**Trial registry information:**

This trial is prospectively registered with ISRCTN (Identifier: ISRCTN11613092), registered on 15 September 2025, 10.1186/ISRCTN11613092.

**Supplementary Information:**

The online version contains supplementary material available at 10.1186/s13063-026-09780-7.

## Introduction

### Background and rationale {9a}

#### Background

Poor mental health and social isolation are increasing among young people [1]. Belonging—feeling connected to and accepted by others through supportive interpersonal relationships has been shown to be an important factor in improving mental health and overcoming loneliness in adolescent populations [2, 3]. A greater sense of school belonging has been associated with a wide array of personal and social benefits including increased psychological wellbeing [4] and reduced instances of school violence, truancy, mental illness and bullying [5–7].

There is, however, a lack of evidence-based interventions addressing belonging, particularly in schools, where socialisation plays a distinct and key role [8]. Evidence-based school interventions could therefore be implemented to promote belonging, making use of an opportunistic target and potentially improving outcomes for adolescents [9].

Digital technologies offer enormous potential for adolescent health promotion and management [10, 11]. The ASP-Belong programme of research has developed *Lina*, a novel intervention whose feasibility has been established through a proof-of-concept prototype [12, 13]. *Lina* uses smartphones to deliver an in-person group experience, in a classroom, that combines immersive storytelling, augmented reality, collaborative gameplay and evidence-based psychotherapeutic techniques to strengthen sense of belonging within the classroom.

*Lina* is the first full-scale version of a new intervention format—Augmented Social Play—which shares the same key ingredients but can be abstracted to different in-person group settings. *Lina* is a multi-session intervention that is delivered within the context of routine school lessons.

#### Rationale

While feasibility has been evidenced through previous work, the effectiveness of the intervention is yet to be established. The current trial is therefore the next step in developing *Lina* and demonstrating its effectiveness on a variety of wellbeing and mental health outcomes, but primarily on sense of belonging. The trial and embedded implementation evaluation will give definitive answers as to how effective this novel intervention is across three national contexts. It will also produce understanding about the best ways to implement the intervention into routine school activities across a number of different contexts, and about young people’s lived experiences of *Lina*, with a particular focus on those who face additional vulnerabilities to poor mental health.

### Explanation for the choice of comparator {9b}

There is no established, widespread school intervention to enhance sense of belonging. Therefore, control classes will continue with standard school activities (i.e. treatment as usual). This comparison offers the most relevant basis for informing educational and policy decisions.

### Objectives {10}

#### Research aim

The primary aim of the trials is to assess the effectiveness of *Lina* in improving children’s sense of belonging within a classroom environment in Portugal, Czechia and the UK.

#### Secondary objectives

Secondary objectives of the study are:

- To test the impact of *Lina* on improving young people’s mental health and wellbeing (more broadly and, specifically, in terms of social anxiety and depression)
- To test the impact of *Lina* on other secondary outcomes including social awareness, loneliness and young people’s stigmatising beliefs.
- To collect the costs of delivering the intervention in comparison to routine school costs and conduct a comprehensive economic evaluation to establish whether *Lina* presents good value for money.

#### Further objectives

Further objectives of the study are:

- To conduct an embedded implementation evaluation to investigate how *Lina* is delivered in secondary schools both within and outside of a trial setting.
- To understand adolescents’ and teachers’ experience of participating in *Lina* and evaluate how the intervention can be adapted to meet the needs of adolescents who are particularly vulnerable to poor mental health.

## Methods: patient and public involvement, and trial design

### Patient and public involvement {11}

Young people were meaningfully involved as co-researchers during the development of the intervention, informing its content, format and acceptability. Ongoing involvement of young people in the design of the trial itself is not planned. During trial dissemination, engagement will focus on participating teachers, communication liaisons and schools to play an active role in scale-up implementation and the promotion of key messages around the trial findings through dissemination activities.

### Trial design {12}

The design of the study is a cluster randomised, parallel group controlled, school-based trial in three countries. Three separate trials will be conducted concurrently in Portugal (PT), Czechia (CZ) and the United Kingdom (UK). While utilising a similar design, these three parallel trials will be independently powered, ethically approved and analysed and will incorporate differences to accommodate local contexts including eligibility criteria—where these differences are planned, they will be highlighted in the protocol.

The intervention provided (i.e. *Lina*) will replace usual lessons within the school term for a period of six lessons and will be facilitated by a teacher in CZ and the UK. In PT, these sessions will be delivered by a psychologist due to national regulatory stipulations on the type of professionals permitted to deliver mental health interventions to children in schools. Outcome data will be collected at baseline and immediately following intervention completion (8–10 weeks post-baseline), and at 6 months follow-up.

Trial data will be analysed separately in the first instance (i.e. country-specific analysis) but will be later pooled to look for overall patterns in the data, particularly for the primary outcome (i.e. class belonging). Pooled data analysis will seek to determine if trial findings are replicated across different European national contexts, and to provide a larger, more varied sample for analysis of effectiveness.

## Methods: participants, interventions and outcomes

### Trial setting {13}

The trial will be conducted within middle schools in Portugal, elementary schools in Czechia and secondary schools in the UK, recruiting whole classes that are comprised of adolescents primarily aged 12*–*13 years. *Lina* has been developed for young people aged 11–14 years and selecting 12–13 years for the modal age in evaluation is a pragmatic decision, which avoids years following major school transitions in PT, CZ and the UK, when we would expect a trajectory of change in mental well-being over the course of the year (i.e. acclimatisation to a new school environment), which could confound the results of the trial.

Schools will be identified through informal conversations, existing networks and local school events. The project team has been actively building relationships with schools since the start of the ASP*belong* programme (September 2023). There will be two types of school involved: (i) flagship schools and (ii) trial schools. Flagship schools will work with the trial team to support networking, and wider engagement, particularly in mainstream media and educational networks. All interested schools will initially be defined as flagship schools. Those flagship schools who are keen to be part of the trial will be supported to become trial schools. Therefore, all trial sites will come from within this network of flagship schools. Schools that become trial schools will therefore already have knowledge of *Lina* through conversations with the research team, public-facing material such as YouTube videos, or presentations during research events and conferences.

We will attempt to purposively recruit trial schools in all three countries, based on their geographical location and other factors of interest, such as level of deprivation. Schools will be invited to join the trial via formal recruitment documents. They will be sent a Local Information Pack which will include the protocol and organisation information document that will act as the data sharing agreement. Recruitment strategies will be tailored to each country’s national context.

## Characteristics of the people who are needed for the trial:

**Table:** 

Characteristic	The people we would expect to see included
Age	● 12–13 years old in the UK ● 12–15 years old in Portugal ● 12–13 years old in Czech Republic
Sex	• Male • Female • Non-binary • Prefer not to say
Gender	Same as above
Race, ethnicity and ancestry	**United Kingdom:** • White (including English, Welsh, Scottish, Northern Irish, British; Irish; Gypsy or Irish Traveller; any other White background) • Mixed/Multiple ethnic groups (including White and Black Caribbean; White and Black African; White and Asian; any other Mixed/Multiple ethnic background) • Asian/Asian British (including Indian; Pakistani; Bangladeshi; Chinese; any other Asian background) • Black/African/Caribbean/Black British (including African; Caribbean; any other Black/African/Caribbean background) • Other ethnic group (including Arab; any other ethnic group) • I prefer not to answer this question **Czech Republic:** • Czech • Ukrainian • Russian • Romani • Vietnamese • Slovak • Other • I prefer not to answer this question **Portugal:** • Portuguese • Brazilian Portuguese • English • Moldovan • Ukrainian • Nepali • Chinese • Other • I prefer not to answer this question
Socioeconomic status	**UK and Czechia:** What is the highest level of education completed by at least one of your parents?
Geographic location	• Birmingham, United Kingdom • Brno, Czech Republic • Torres Vedras, Portugal In CZ and the UK, schools will be recruited near to the respective research bases (University of Birmingham and Masaryk University), which both reflect urban areas, with high numbers of relatively disadvantaged schools
Other characteristics relevant to the trial	• Proportion of students receiving 1:1 support by school staff (to determine level of special educational needs in the population) • Proportion of students who have previously attended another school (to determine level of mobility of students between schools in the population)

### Eligibility criteria for participants {14a}

Eligibility criteria for student participants will be consistent across the three trials, to ensure that the sample is comparable and trial data from the three trials can be pooled.

#### Inclusion criteria

Schools will be included as “trial schools” if they:

- Are a school in the UK with grade 8 students (aged 12–13)
- Are a school in Portugal with grade 8 and 9 students (aged 12–15)
- Are a school in Czech with grade 7 students (aged 12–13)
- Agree to support the delivery of the *Lina* as part of routine school activities in the 2025/26 academic year
- Agree to the process of randomisation, with the understanding that classes which are randomly allocated to the control group will have to wait until the 2026/27 school year to implement the *Lina*

Individual young people will be included in the trial if:

- They are 12–13 years old in the UK
- They are 12–15 years old in Portugal
- They are 12–13 years old or are classes with 12–13 years old in Czech Republic
- They are part of a class with similarly aged students
- Assent to the collection of self-reported questionnaire data at key timepoints through the trial (baseline, 8–10 weeks post baseline, and 6 months baseline)
- They have a parent/legal guardian who has provided written informed consent for collection of the young person’s personal data (only in CZ)

The variation across the age eligibility criteria in each trial country is reflective of the differences in the educational systems, grade structures and ethical requirements in the UK, Czech Republic and Portugal. In the UK, year 8 students are almost uniformly 12–13 years old, while in Czechia and Portugal there is a high rate of grade retention for students. While the modal age for the intervention is still 12–13 years, the higher number of older children in the corresponding grades in the trial is informed by a pragmatic approach that prioritises inclusion and ecological validity across the vulnerable population.

From this trial sample, a subsample of young people will be invited to attend an interview or focus group as part of the study’s implementation evaluation and/or exploration of lived experiences of *Lina*. The specific inclusion criteria and sampling methodology for this aspect of the study is outlined under Recruitment {20}.

#### Exclusion criteria

Young people will be excluded from the trial if:

- They have a parent or guardian who explicitly requests for them to be opted out of the trial

Even if a young person, or a parent or guardian requests for the young person to be excluded from the study and they “opt out” of participation, the young person will still receive the *Lina* intervention as part of routine school activities. The “opt out” refers to the completion of the trial outcome measures only.

### Eligibility criteria for sites and those delivering interventions {14b}

Schools will be included as “trial schools” if they:

- Are a school in the UK with grade 8 students (aged 12–13)
- Are a school in Portugal with grade 8 and 9 students (aged 12–15)
- Are a school in Czech with grade 7 students (aged 12–13)
- Agree to support the delivery of the *Lina* as part of routine school activities in the 2025/26 academic year
- Agree to the process of randomisation, with the understanding that classes which are randomly allocated to the control group will have to wait until the 2026/27 school year to implement the *Lina*

Young people will be included if they:

- Are aged 12–13 years old in the UK
- Are aged 12–13 years old, or in a class among other students aged 12–13 years old in Czechia
- Are aged 12–15 years old in Portugal

### Who will take informed consent? {32a}

Schools signing up to the trial will have already made the decision that the intervention does not differ significantly enough in terms of risk or content from standard school activities to indicate that any consent will be required from individual students in order for them to partake in the *Lina* experience. This is critical to the successful delivery of the intervention as *Lina* requires the inclusion of whole classes of young people.

Thus, for the purpose of the trial, consent will specifically mean consent for completing trial measures and the contribution of trial data.

Therefore, in PT and the UK an “opt-out” method of parental consent will be utilised. This means that schools, in collaboration with the trial team, will inform parents and students about the trial fully before any baseline measures are taken. The study team will work with schools to identify the best way to reach all relevant parents/carers with information about the trial, particularly about the types of data we will be collecting and the questions we will be asking as part of the standardised outcome measures. The trial team will also endeavour to organise information events at each school to give further information about the trial and outline what is involved so that adolescents and parents/carers are fully informed. For example, in many schools this will include researchers attending a parents’ meeting when we will talk to all attending parents about the study and signpost where parents/carers can find out more information about the research and be given the opportunity to ask any questions about the research, before enrolment in the study takes place.

Therefore, all young people within a trial classroom will be enrolled in the trial unless an express request has been made by the student and/or their parent/carer to not be involved. In such cases, young people will still take part in *Lina* as part of their usual school activities, but no trial outcome data will be collected from them, and they will not be included in the trial sample. Such “opt-out” consent has been used widely in school-based studies of this type (e.g. DEER study [14]) to improve recruitment, reduce researcher burden and improve engagement and information-giving with participants and parents/carers.

Trial schools can request that consent procedures are altered or changed based on parent feedback, and these decisions will be made on a school-by-school basis.

In CZ, local research ethics requirements preclude the possibility of out-out consent. In this country, parents/legal guardians will be asked to provide written consent for their children to participate, which must be in place prior to the commencement of data collection.

In all 3 countries, young people will also complete a survey consent form at the point of baseline data collection, to document that the study has been explained to them, they have had the opportunity to ask questions and they adequately understand what information they are expected to give, and they assent to their provided data being used in this way.

For students and teachers who are invited to participate in the qualitative interviews and focus groups that make up part of the implementation and/or lived experience evaluation, full informed consent will be collected before any data is collected.

All participant information sheets and consent forms are provided in Appendix 1.

### Additional consent provisions for collection and use of participant data and biological specimens {32b}

All consent provisions have been mentioned under heading *32a*.

## Intervention and comparator

### Intervention and comparator description {15a}

#### Description of the intervention

The intervention under investigation *Lina* is a newly developed software-led program with accompanied in-person reflection designed to enhance students’ sense of belonging through a blended approach of interactive gameplay facilitated by smartphones in a classroom setting. All necessary equipment, including smartphones, routers, headphones and paper-based materials will be provided by the trial team to the class.

The intervention is primarily user-driven but will be guided by the classroom teacher (or a psychologist in the case of PT). Additionally, a researcher will be present during every session to make observational notes and support usage, where required. The intervention consists of six sessions. Four of these are “episodes” of the *Lina* story and will be delivered on a provided individual smartphone, while two will be “analogue” sessions, which are structured and interactive sessions within the classroom to enhance the sense of belonging and embed the psychotherapeutic principles inherent in the intervention. In CZ and the UK, it is typically teachers who will deliver the analogue sessions, in line with detailed lesson plans and provided materials. In PT the psychologist members of our team will deliver these sessions, in line with national legislation.

The order will be as follows:

Lesson 1: *Lina* episode 1.

Lesson 2: *Lina* episode 2.

Lesson 3: Analogue session 1.

Lesson 4: *Lina* episode 3.

Lesson 5: *Lina* episode 4.

Lesson 5: Analogue session 2.

During the smartphone-based sessions, each student plays a character in a fictional class. The four *Lina* episodes follow the story of a girl called Lina, a fictional classmate who abruptly left the school. Lina had been secretive, and at times, Lina’s behaviour seemed unusual or difficult to understand. The class collectively decides to uncover what happened to her. Players examine objects Lina left behind using augmented reality, revisit shared memories and piece together the story. By the last episode, they learn that Lina has been caring for her mother, who struggles with a poor mental health (low mood, guilt) that has profoundly affected their daily lives. Lina and her mother have moved to live with Lina’s aunt, allowing her mother to receive support while Lina returns to her previous school.

Gameplay emphasises collaboration and problem-solving, both individually and in varying group sizes. Students (and occasionally the teacher) work together to solve puzzles, exchange information, and help one another progress. Group compositions are intentionally mixed throughout the sessions to ensure every student interacts with their peers. Each *Lina* episode ends in a 5-min out-of-character period of reflection, still led by the smartphone.

Using Lina’s story, the teacher- or psychologist-led analogue sessions help students process the narrative and deepen their understanding of the themes explored (e.g. perspective taking, empathy, impact hidden challenges). The group will reflect on the overall experience, their classroom dynamics and their sense of belonging within the group. This will enable contextualisation of the main learning themes.

All necessary materials for these reflection sessions will be supplied by the trial team.

#### Description of the comparator

Control classes will engage in standard school activities/routine lessons and will be provided the intervention in the beginning of the next school calendar year.

### Criteria for discontinuing or modifying allocated intervention/comparator {15b}

As the trial allocates intervention at the classroom level, discontinuation or modification of the allocated interventions in the trial may occur at the following levels:

1. Individual student-level: A student may postpone or terminate active participation in the intervention if (a) excused by the teacher or school administration in accordance with the school’s policies or procedures or (b) severe safeguarding concerns related to the intervention as determined by the Trial Steering Committee (TSC)
2. Class-level: An intervention class session may be postponed or terminated early due to (a) operational constraints such as emergency closures, strikes or staff shortages experienced by the school or (b) severe safeguarding concerns related to the intervention determined by the TSC

### Strategies to improve adherence to intervention/comparator {15c}

We will develop Standard Operating Procedures (SOPs) around consenting procedures, data collection and management and intervention delivery to ensure fidelity and adherence to the protocol. All implementation researchers will fill out session logs to introduce a standard monitoring framework for data quality.

Although we do not have any target tools to measure adherence and fidelity specifically, we will be monitoring fidelity and adherence to the intervention recording the number and content of each session using observation forms which will be filled out by researchers facilitating *Lina* lessons.

### Concomitant care permitted or prohibited during the trial {15d}

As this is taking place within routine classes in schools, students and teachers will continue to receive all usual educational and wellbeing support delivered by schools. Given the vulnerable status of young participants in the trial, they will not be excluded from accessing additional interventions during the trial period. Information on the use of any additional support services availed of by students as a result of the trial within schools will be captured through trial assessments (e.g. Health Economics Inventory forms) and reviewed in the analysis to assess the cost of delivering the intervention in the schools.

### Ancillary and post-trial care {34}

This trial has been identified as a low-risk, school-based psychosocial intervention so we do not anticipate participants would require ancillary or post-trial care. In the event that participants do require additional support, they will continue to have access to the usual educational and mental health support available within their schools and we will follow school safeguarding policies and procedures at all times. The study is covered under the University of Birmingham’s standard research indemnity scheme for participants.

### Outcomes {16}

The trials will collect information from participants on a range of mental health, social and economic evaluation-related outcomes including sense of belonging, symptoms of depression, social anxiety, loneliness, quality of life, stigma, social awareness and resource use.

#### Primary outcome

The primary outcome will be sense of belonging/class climate as measured by the Delaware School Climate Survey- Student (DSCS-S) measured post intervention (8–10 weeks) and at follow-up (6 months). This is a 23-item student-report scale to measure the sense of belonging an individual feels within a classroom context [15]. The scale is comprised of five subscales: Teacher-Student Relationships; Student–Student Relationships; School Safety; Fairness of the Rules and School Liking [16]. Items are rated on a Likert scale, with negatively worded items reverse-scored. Subscale scores for Teacher–Student Relationships, Student–Student Relationships, School Safety, Fairness of the Rules and School Liking are computed as the mean of their respective items. The overall school climate score is calculated as the mean of all 23 items and analysed as the mean difference in total or subscale scores between Lina and control groups from baseline and post-intervention scores (controlling for baseline score and clustering). The DSCS-S is justified for measuring this outcome as it demonstrates strong psychometric validity and reliability in Portugal [17], Anglophone contexts, e.g. USA [16], and in Eastern European settings, e.g. Serbia [18] along with widespread international use in low-resource settings. The wording of the items, in all three languages, has been adapted slightly to ask about the class climate, as opposed to the overall school climate. This is in order to make the measure sensitive to the climate of the specific classroom in which the intervention was delivered.

#### Secondary outcomes

In line with the developing *Lina* theory of change, secondary outcomes were selected to assess constructs anticipated to be affected by *Lina.* General wellbeing may be increased via increased belonging or other aspects associated with *Lina* such as exposure to the arts. Social anxiety and depression were selected as symptoms of mental distress that are both some of the most commonly experienced in adolescents, and (in the case of *social* anxiety) most likely to be positively impacted by *Lina*. Social awareness represents an area of psychological resources expected to be positively impacted by *Lina.* Loneliness is sometimes conceptualised as the counterpoint to belonging and is expected to be positively impacted by the positive class environment created by *Lina.* Finally, the *Lina* story touches directly on mental health issues in a way designed to reduce stigma. Measure selection was strongly influenced by availability of prior validation with young people in PT (where specific regulatory constraints exist around measures that can be used with young people in school settings), and by availability of data on sensitivity to change. Thus, the following student-report measures were selected. For all questionnaires, the total score (sum of all items) is the unit of analysis. For each outcome, the metric for analysis will be the mean difference in total or subscale scores between Lina and control groups at post-intervention, adjusted for baseline score and clustering via linear mixed methods models.

- WHO-5 Well-being Index is a 5-item measure of general well-being that has proven reliability and criterion validity among adolescents in the UK, Portugal and Czech Republic as part of a large cross-national validation study [19]. Scores are transformed to a score from 0 to 100, with lower scores indicating worse well-being [20].
- Patient Health Questionnaire-8 is an 8-item screening tool designed to assess the presence of depression symptoms [21]. PHQ-8 has strong equivalence with PHQ-9, has been tested for reliability and validity in 27 European countries—including the UK, Portugal and Czech Republic [22] and has demonstrated high internal consistency when administered with adolescents from vulnerable settings [23]. The total score can range from 0–24, with a score of ≥ 10 indicative of clinical-level depression [21].
- Social Anxiety Scale for Adolescents (SAS-A) is an 18-item questionnaire measuring adolescents’ social anxiety experiences in the context of peer relations [24] SAS-A has proven reliability and validity for Portuguese adolescents [25] and has been used in school-based screening of adolescents across France and Spain [26–28] with acceptable internal consistencies and validity. It contains three subscales: Fear of Negative Evaluation, Social Avoidance and Distress-New and Social Avoidance and Distress-General. Total score ranges from 0 to 90 with a higher score indicating a higher level of social anxiety [24].
- The 4-item University of California Los Angeles (UCLA) Loneliness Scale is a revised and shorter version of the full UCLA tool (20 items) [29]. The full UCLA scale demonstrated modest internal consistency in the assessment of loneliness in children and adolescents [30]. This version of the UCLA has been recommended by the Office of National Statistics in the UK [30, 31] as a strong measure of loneliness in youth aged 10–15 years, following adaptations. The tool has been translated and validated in Portugal and Slovakia with a modest internal consistency [32, 33].
- Social-Emotional Learning Edition of the Strengths and Difficulties Inventory System [brief version] (SSIS SELb) Social Awareness subscale is a 4-item assessment that evaluates the social-emotional skills of children and adolescents in relation to their social awareness. It measures how much students take into account others’ point of view, particularly those from different backgrounds [34]. SSIS SELb was translated and validated for use among school-going adolescents in 6 European countries including Portugal with a strong measurement for reliability and structural validity, supporting its use for international research with appropriate adaptation and translation [34].
- Peer Mental Health Stigmatisation Scale-Revised (PMHSS-R) Stigma Awareness subscale is 5-item assessment of individually held stigmatising beliefs. It is answered on a 5-point Likert scale of agreement [35]. PMHSS-R has been developed in Ireland [35] and validated in European contexts (Spain) [36] but there is no country-specific validation for the UK, Portugal and Czech Republic. However, at the time of drafting this protocol, PMHSS-R is currently the only validated tool that assesses both awareness and agreement of stigma and was considered justified in use for measuring these outcomes in the trial.
- EQ-5D-Y-3L is a child-friendly questionnaire to describe young peoples’ health across five dimensions: Mobility; Looking After Myself; Doing Usual Activities; Having Pain or Discomfort; and Feeling Worried, Sad or Unhappy [37]. Each dimension has three levels of response. The tool has been translated in Czech [38] and Portuguese [39] and demonstrates strong psychometric evidence for validity and reliability with adolescents in the UK [40] and among Portuguese speakers [39].
- Child Health Utility-9D (CHU-9D) is a self-report measure of health-related quality of life suitable for 7- to 17-year-olds. It consists of 9 dimensions each measuring a different domain (worried, sad, pain, tired, annoyed, schoolwork, sleep, daily routine and activities). Each domain has 5 levels of severity. The CHU-9D has been locally translated for administration among young people in Portugal, Czech Republic [41] and the UK and demonstrated modest internal consistency and validity among children, adolescents and young adults within the UK and Portuguese speakers [39].

#### Resource use data

We will collect three groups of data for resource use in the economic evaluation.

- First, we will collect data from teachers/psychologists on their time spent preparing and delivering *Lina* and usual sessions. The data will be collected for three periods, i.e. at baseline (with 3 months recall), the end of 8–10 weeks intervention (with 8–10 weeks recall for control group and weekly data collection for intervention group after each *Lina* session), and 6 months follow-up (with 6 months recall). In addition to time spent by teachers/psychologists, we will also collect data on other professionals’ time spent (e.g. administrative staff, teaching assistants) on delivering *Lina* or usual sessions. We developed four Health Economics Inventory forms for data collection on time spent by teachers/psychologists and other professionals.
- Second, our research team will collect data on the quantity and costs of equipment used for delivering *Lina* sessions, including smartphones, routers, headphones, and paper-based materials.
- Third, we will collect data on time spent by teachers/psychologists and other professionals (e.g. administrative staff, teaching assistants, counsellors) providing additional mental health/wellbeing support to students as well as managing students’ disruptive behaviours. The data will be extracted from school records for the period from 3 months before baseline till the end of 6 months follow-up.

#### Qualitative data

Following completion of *Lina*, focus groups and interviews will be conducted with both student participants and teachers who were involved to collect implementation and experiential data.

Focus groups and interviews will be audio recorded and anonymised transcripts will be the data subject to analysis. For the purpose of data analysis, transcripts for 3 interviews, 1 focus group with young people and 1 teacher focus group from each country will be translated initially and the UK, CZ and PT teams will collaboratively devise a coding framework. This framework will then be used to conduct first-level data analysis separately in each country. Data will then be collated and analysed together by UK, CZ and PT researchers collaboratively.

#### Implementation evaluation data

To further understand how *Lina* would be implemented as part of routine school activities, and to inform dissemination and further implementation strategies, an embedded implementation evaluation will be conducted both during the trial and after it is completed. The implementation evaluation will draw from multiple data sources, using a mixed methods design, to get a full insight into the barriers and facilitators that would affect *Lina* delivery and learn how to increase reach and improve roll out. This evaluation will be conducted across trial schools in all three countries.

The primary data sources will be:

- *Questionnaire measures completed by teachers*—Teachers from intervention classes will complete questionnaires on feasibility (“Feasibility of Intervention Measure”), acceptability (“Acceptability of Intervention Measure”) and appropriateness (“Appropriateness of Intervention Measure”) [42] after each episode/session of *Lina* to give feedback on each session. They will then also complete these measures at post-intervention timepoint to feedback on the intervention as a whole.
- *Implementation data provided by teachers*—Teachers from intervention classes will answer three broad questions on *Lina* “what went well?”, “what didn’t go well?” and “what could have gone better?”. These questions will be asked by the researcher after each session of *Lina* for immediate feedback on the delivery of that session. Teachers will also answer these questions, retrospectively, at the end of the intervention to give feedback on *Lina* as a whole.
- *Structured observations*—A researcher will be present to conduct structured observations of all *Lina* sessions. *Focus groups (teachers and psychologists)*—Following the completion of *Lina*, teachers will be invited to participate in focus groups to elaborate on their experience of implementation, and give interactive feedback on potential strategies to facilitate implementation.
- *Focus groups (young people)*—Students from intervention classes who experienced *Lina* will be invited to participate in focus groups where they can elaborate on their lived experience of using the intervention and how its delivery could be improved.

#### Data collection timepoints

The baseline measures will be completed after the class has been randomised, but prior to the beginning of the intervention.

Baseline measures will be collected *at least* 1 week before the first session of *Lina*. It is most likely that baseline data will be collected 2 weeks before the first *Lina* session, therefore allowing enough time to collect data from all students in the class.

Post-intervention measures will be collected between 8 and 10 weeks from the date of baseline assessments (allowing a 2-week time window, while accounting for the instances where classes hold multiple intervention sessions in a week). Six-month follow-up measures will be collected within 4 weeks (either side) of the date of six whole months since baseline assessments.

Measures will be completed as part of the routine school day, within a class session. If a participant is absent from school, or data is missing for any reason, then the research team will have 3 weeks from the data collection time point to collect that data.

All trial assessments will need to take place within the 2025/26 academic year. In the rare case that schools or classes are delayed in their delivery of *Lina* and final follow-up falls outside of the summer academic term then follow-ups may need to take place sooner than 6 months post baseline.

### Harms {17}

Participation involves low expected risk, e.g. emotional distress during data collection, safeguarding disclosures during the intervention or minor physical injury during classroom activities. Risks will be mitigated through the use of age-appropriate measures, clear information about voluntary participation and withdrawal and clear Standard Operating Protocols to guide researchers on how to identify and manage risks sensitively when working with adolescents, and delivery in schools with existing safeguarding support. Researchers will monitor participants throughout all activities and may pause or stop data collection if concerns arise. Clear procedures are in place to guide measured responses to any harm identified or reported and escalate concerns to school safeguarding leads where required. All adverse events are recorded (expected, unexpected, related or unrelated), with serious events reported promptly to the research leadership and ethics committees within 24 h.

### Participant timeline {18}

**Table:** 

	**Trial period**			
	**Enrollment**	**Post-randomisation**		
**TIMEPOINT**^**b**^	*** − t***_***i***_** to 0**	***t***_***0***_*** (Baseline)***	***t***_***1***_*** (Post-intervention)***	***t***_***2***_*** (6 months)***
**ENROLLMENT:**	X			
**Eligibility screen**	X			
**Allocation/randomisation**	X			
**Informed consent**		X		
**INTERVENTION/COMPARATOR:**				
***Lina* lessons**		X ––––––––- > X		
**Routine school lessons**		X ––––––––- > X		
**ASSESSMENTS** **(YOUNG PEOPLE):**				
**Demographics**		X		
**Delaware School Climate Survey- Student**		X	X	X
**WHO-5 Well-being Index**		X	X	X
**Patient Health Questionnaire-8**		X	X	X
**Social Anxiety Scale for Adolescents (SAS-A)**		X	X	X
**University of California Los Angeles (UCLA) Loneliness Scale**		X	X	X
**Social-Emotional Learning Edition of the Strengths and Difficulties Inventory System [brief version] (SSIS SELb)**		X	X	X
**Peer Mental Health Stigmatisation Scale-Revised (PMHSS-R)**		X	X	X
**Health-related Quality of Life EQ-5D-Y-3L**		X	X	X
**Child Health Utility-9D (CHU-9D)**		X	X	X
**Intervention (*Lina*) feedback**			X	
**Interviews and Focus Groups**			X	
**ASSESSMENTS (TEACHERS):**				
**Demographics**		X		
**Health Economics Resource Inventory**		X	X	X
**Implementation evaluation form (Feasibility, Acceptability, Appropriateness)**			X	
**Intervention (*Lina*) feedback**			X	
**Focus Groups**			X	

### Sample size {19}

#### Size of trial sample

Based on an assumed small to medium effect size (Cohen’s *d* = 0.4) based on school mental health meta-analyses [43], and a conservative Intraclass Correlation Coefficient (ICC) of 0.1 [44], which takes into account the clustering at the class and school level, and a design effect of 2.7, 40 classes per country will provide 90% power (alpha = 0.05).

The analysable trial sample assumes an average cluster size of 18 (a total of 720 participants per country). To allow for an anticipated drop-out rate of 20%, an average cluster size of 22 will be recruited at baseline (i.e. average 22 adolescents per classroom), thus giving a total sample size of 880 per country. The cluster size of 22 at baseline takes into account the variability in class sizes within and across each country, and expected number of participants who opt out of the study.

#### Size of interview and focus group sample

For the post-trial interviews and focus groups it is predicted that there will be a maximum of 160 young people per country invited to participate in the evaluative qualitative work (up to 120 young people invited to a focus group, and 40 young people invited to be interviewed).

For teachers, there is predicted to be 20 teachers per country eligible to be interviewed or invited to a focus group.

### Recruitment {20}

#### Sampling technique

In order to recruit 40 classes per country, we predict that around 10 schools per country will need to be recruited, with each contributing an average of 4 class cohorts. We anticipate there will be variations in the number of class cohorts available within individual schools across countries.

This will represent an overall opportunistic sampling method, making use of schools who have indicated an interest in the *Lina* and have agreed to become a flagship school. However, we will aim to purposively recruit schools with higher levels of deprivation as deprivation is linked to greater risk of poor mental health.

In PT, the percentage of Free and Reduced Cost School Meals will be used as an indicator of deprivation. Young people who come from the lowest economic socioeconomic status have free lunches (they are listed as A in the school support system) and young people from low, but slightly higher, socioeconomic status families pay 50% of the lunch (and they are listed as B in the school support system).

We will aim to recruit schools with the highest numbers of students listed in category A for Free and Reduced School Meals.

In CZ and the UK, schools will be recruited near to the respective research bases (University of Birmingham and Masaryk University), which both reflect urban areas, with high numbers of relatively disadvantaged schools. Although priority will be given to pragmatic considerations (ease of access, eagerness of school to participate), local knowledge will be used to preferentially shift recruitment towards more disadvantaged schools. In the UK, this is reflected by schools having a higher-than-average proportion of students meeting the low socioeconomic status criterion for additional school funding from the government (“pupil premium”), as available on government websites (≈ > 30%). In both countries, it is reflected by relatively high levels of ethnic minority students, as ascertained based on local knowledge.

#### Recruitment of schools

Schools will be identified, approached and recruited across the three countries using a number of different strategies that are adapted to the national context. In general, the trial team will make use of existing networks, conferences, seminars and learning events attended by headteachers and teachers to promote the study, and assess interest. Those schools that want to support the study will be offered to become a “flagship school” to support in the networking within educational networks about the intervention and the objectives of the trial. Flagship schools will be given the opportunity to become a trial school (i.e. a trial site from which we recruit classes and participants from), but this will not be mandatory.

#### Sample identification

Once a school has agreed to be a trial school then the trial team will support them to identify whole classes that have students that meet the eligibility criteria. We will work with schools to assess how best to deliver *Lina* within the routine school day, i.e. identify a specific subject or class that could integrate the intervention and be part of the regular school day. As an example, in the UK it is likely that the intervention will be delivered as part of Personal, Social, Health and Economic (PSHE) lessons which covers a range of topics related to personal development. However, schools can individually choose where they think *Lina* best fits within their curriculum.

The variation of subjects and lesson content both across schools and across countries means that the trial team will take a pragmatic approach to delivery and support the schools in making tailored decisions. It is recommended to schools that each of the six *Lina* sessions is delivered weekly for 6 weeks within a single term. However, due to the pragmatic nature of the trial, and the understanding that schools (both within and between countries) structure lessons in different ways, schools may choose to deliver the sessions at different frequencies or across shorter or longer time periods (e.g. some schools may choose to deliver two sessions of *Lina* a week for 3 weeks). The variation in delivery will be collected as the observational data and form part of the analysis. Irrespective of the intervention delivery, follow-up outcome measures will be collected at 8–10 weeks after baseline.

#### Interview and focus group sampling

Purposive sampling will be used to identify individuals of interest to participate in the planned post-trial interviews and focus groups (that make up part of the implementation evaluation and exploration of lived experiences).

For young people, individual interview data will be collected from particularly vulnerable students, who want to talk about *Lina* (i.e. have something to say), and have characteristics that make them more likely to have relatively extreme (good or bad) experiences of *Lina*. Focus group data will be collected from students who want to talk about *Lina*, for example because they have had a particularly good or bad experience of *Lina.* In the UK, the vulnerable students for interviews will be defined by three separate criteria: (i) recipient of pupil premium, (ii) having special educational needs and disabilities (SEND) and (iii) having experienced “notable social experiences” e.g. negative experiences like bullying and/or positive social experiences such as being part of an extracurricular club or society outside of school. Students will have the opportunity to self-identify with (any of) these characteristics and teachers will also be asked to recommend students. In PT and CZ, students for interviews will be identified based on discussion with teachers. In all countries, students will be given the opportunity to self-select for focus group participation, and students may also be recommended by teachers or by observing researchers.

For teachers—all teachers who facilitated the use of *Lina* will be invited to attend an interview or focus group.

## Assignment of interventions: randomisation

### Sequence generation: who will generate the sequence {21a}

#### Randomisation scheme

The trial will employ a cluster randomised design, with class being the unit of randomisation. Whole classes will be randomised to either the *Lina* arm or the control group arm.

The trial statistician at Queen Mary University of London will use STATA to randomise a list of classroom IDs to either intervention or control conditions. IDs will then be assigned to classes in the order in which they are enrolled.

Classes within schools will be randomised at the same time (where possible) and the school and individual teachers will be informed of their allocation by the local research assistant or the trial manager.

### Sequence generation: type of randomisation {21b}

Randomisation will be stratified by country, and then stratified by blocks of 2 or 4. The randomisation scheme may be amended as and when the full list of participating trial schools is known. Any future amendment to the randomisation scheme will be there to ensure that there is at least one class in the *Lina* arm per school (important for school retention) and to ensure that there are comparable numbers of participants randomised to each of the trial arms.

Randomisation will occur only when: schools have agreed to be part of the trial; appropriate classes that meet the inclusion criteria have been identified; adolescents have agreed to the study and all students have been allocated to the class. Once these terms have been met then randomisation can be triggered. Randomisation will occur before participant baseline data collection. The allocation ratio will be 1:1.

### Allocation concealment mechanism {22}

Due to the nature of the trial, allocation concealment can only be maintained at the level of data collection. To ensure allocation concealment, password-protected randomisation allocation lists generated by the statistician will be maintained in Excel files and shared only with the Trial Manager and unblinded researchers in each trial team responsible for intervention scheduling. Passwords for the excel sheets will be communicated via a secure separate channel and deleted afterwards to prevent accidental disclosure to blinded researchers responsible for facilitating trial assessments. Logs containing any data linking classroom-level information to concealment will be restricted to unblinded researchers until the end of the trial assessments (e.g. post-intervention and 6-month follow-up).

### Implementation {23}

As the unit of randomisation in this trial is the classroom, all students within participating classrooms will be enrolled into the trial, provided they have met the relevant consent criteria. The procedure is listed below:

1. The trial team in each country will be responsible for enrolling eligible classrooms within recruited schools.
2. A list of classroom IDs will be produced and shared with the Trial Statistician who will generate the allocation sequence using block randomisation (blocks of two and four) to assign classrooms to intervention or control arms. IDs will then be assigned to classes in the order in which they are enrolled.

## Assignment of interventions: blinding

### Who will be blinded {24a}

#### Blinding

Due to the nature of the intervention under investigation and the cluster design of the trial, blinding participants (and teachers) to their allocation will not be possible. Baseline data will be collected post-randomisation, but prior to the start of the intervention. Participants in the class will not be informed of allocation prior to baseline data collection (although teachers will be aware due to pragmatic considerations around lesson planning, for example).

In the UK trial, researchers facilitating the baseline, post-intervention and 6 months follow-up will be blinded to allocation where possible.

Due to researcher capacity in Portugal and Czechia, blinding will not be possible at any of the post-intervention follow-ups (i.e. T1 and T2).

The statistician and health economist will be unblinded to allocation at the time of analysis. Neither analyst will have involvement in data collection, intervention delivery nor allocation concealment and their roles will begin once the database is locked and the randomisation sequence is revealed. As recent literature suggests, there is limited benefit of blinding the statistician at data analysis if allocation concealment is maintained during data collection [45]. To further minimise the risk of analytical bias, the statistical analysis plan (SAP) will be finalised and signed off by the Trial Steering Committee before database lock and commencement of analysis, specifying all primary and pre-planned sensitivity analyses.

### How will be blinding be achieved {24b}

Blinded researchers will be determined prior to the start through the Trial Delegation log. To avoid any unblinding, efforts will be made to ensure that unblinded researchers are not responsible for coordination, allocation information will be stored separately from assessment material, will be password-protected and masked in any analysis datasets until the primary analysis is completed.

### Procedure for unblinding if needed {24c}

Unblinding researchers to participant allocation may occur in the interest of participant safety e.g. if the knowledge of the participant’s allocation is necessary for the appropriate management of an adverse event or severe adverse event. Any such request would be formally documented in a trial file note, and reported to the Principal Investigator and Trial Manager.

## Data collection and management

### Plans for assessment and collection of outcomes {25a}

All outcome measures used have been validated for use with adolescents. The list of outcome measures was decided upon thorough consultation with the Trial Management Group (TMG) and a panel of young co-researchers (see heading 12 for full list of outcome measures).

#### Data collection for outcome measures

Electronic case report forms (e-CRF) will be created to collect socio-demographic data and outcome data (separate versions for each timepoint) (these can be accessed on the ASP*belong* Open Science Framework project page [46]). The e-CRFs will be hosted on smartphone-compatible software that will link to the online trial database (REDCap) through a unique participant number. At baseline, a record will be created in the database for each participant. Following baseline data collection, an individual URL will be created by the REDCap software for each participants’ data row. At post *Lina* and follow-up data collection timepoints, a bespoke application (built by the project team) will open the e-CRF linked to the correct URL when the corresponding participant number is entered. Participants will complete the measures on the smartphones provided by the study team (the same devices used to deliver the intervention). In the event of technical challenges with electronic data entry (e.g. Wi-Fi issues), paper forms will be used to complete the assessment.

e-CRFs will be completed as part of the routine school day, within a class session. They will be collected from all consenting students within that class at the same time, facilitated by the class teacher or a psychologist (PT) and a researcher (UK, CZ), who will offer support where needed around understanding questions in a consistent manner within each country context (i.e. explaining certain terminology within item wording that students query). If a participant is absent from school, or data is missing for any reason, then the research team will have 3 weeks from the data collection time point to collect that data.

### Plans to promote participant retention and complete follow-up {25b}

We will not be collecting any individual-level contact information for participants except where necessary to coordinate data collection for people actively opting into interviews and/or focus groups. Efforts for participant retention and mitigating attrition will therefore be on a school-level. We have worked on establishing strong relationships with schools and teachers through regular communication, several networking opportunities, and emphasising a strong sense of collaboration. Further, we will be flexible with scheduling lessons to accommodate the academic calendar/exam time, or any other school events.

No data will be collected from students who withdraw themselves or whose parents withdraw them from the trial. Furthermore, in the event that a student was to leave the school or be transferred to a different class within the same school during the intervention, they would cease to receive the intervention, and no further data would be collected from them.

### Data management {26}

The primary mode of data capture will be through electronic Case Report Forms (e-CRFs) administered on smartphones via REDCap (Research Electronic Data Capture) which is a secure, web-based system designed for building and managing research data. If REDCap is unavailable (e.g. device malfunction, connectivity issues), paper Case Report Forms (CRFs) will be used. Paper CRFs will be entered into REDCap at the earliest opportunity, with the paper copy stored securely at each site in a locked cabinet and stored separately from any identifiable enrolment information. Each participant survey will be linked to a unique participant ID (PID), and the follow-up assessments are linked to it. Paper-based data can be transferred between individual sites (e.g. schools) to the central site but will never leave the central site (e.g. University of Birmingham in UK, Masaryk University in CZ and Académico Torres Vedras (ATV) in Portugal). Access to the REDCap portal will be granted to designated and trained researchers at each site for the manual data entry of paper CRFs into the online portal. Site leads and researchers will be responsible for first-level quality control of the data entry and checks. The trial manager will oversee routine monitoring and any data errors in the database will be queried and resolved with the relevant site team. Once data is entered into REDCap, data exports can be produced through reports for data verification and analysis by the Trial Statistician.

### Confidentiality {33}

The trial will comply with the requirements of the UK Data Protection Act 2018, as standard, with regard to the collection, storage, processing and disclosure of personal information and will uphold the Act’s core principles throughout the study, and be UK General Data Protection Regulation (GDPR) compliant. As all trial data (from all three countries) will be stored in the UK, the relevant national legislation of the UK will be followed:

All data will be confidential at the point of collection, linked to participants via unique ID numbers. Identifying information (participant full names and contact details) will only be collected for participants (students and teachers) in the qualitative and implementation evaluations, and only when necessary (e.g. to arrange data collection with a student, which may otherwise be arranged via the class teacher). Personal information will be stored in password-protected spreadsheets at the local research site (University of Birmingham, Masaryk University, Académico Torres Vedras) and linked to research data via the unique ID.

Interviews and focus groups (qualitative and implementation evaluations) will be audio recorded and these data will be potentially identifying. Recordings will be stored securely at each research site until transcription, when any potentially identifying information will be removed (transcripts will be anonymous). Following verification of the transcripts, the audio data will be deleted. Only anonymous data will be shared across research sites for analysis.

## Statistical methods

### Statistical methods for primary and secondary outcomes {27a}

#### Statistical analysis

Data for the three trials will be analysed separately (by country), before later being pooled for overall analysis.

Participant level socio-demographic data and cluster level (classroom) data will be summarised at baseline for both arms of the trial.

The primary outcome is the Delaware Class Climate score at the end of *Lina* delivery (8–10 weeks post baseline and follow-up). This will be compared between *Lina* and control groups using a linear mixed effects model with a random effect for classroom and school, allowing for the clustering of children, within classes, and within schools. The model will include baseline level of the Delaware Class Climate as a covariate, as well as demographics (that are known to affect outcome). The analysis will use intention to treat by including all participants in the arm to which they were randomised, whether or not they completed the *Lina* programme and including all students in the analysis by using multiple imputation. We will set out in the statistical analysis plan (SAP) how missing data will be handled, including planned sensitivity analyses.

Secondary outcomes are WHO-5, PHQ-8, SAS-A, UCLA Loneliness Scale, SSIS SELb, PMHSS-R, EQ-5D-Y-3L and CHU-9D all measured at post-intervention (T1) and 6 months post baseline (T2). Each variable will be analysed using a generalised linear mixed effects model with a link function appropriate to the outcome distribution, with random effects for classroom and school. All models will include the baseline value of the outcome.

#### Economic evaluation

We will conduct an economic evaluation alongside the three trials aiming to assess the cost effectiveness of Lina in comparison with usual lessons from a school perspective. The time horizon for the evaluation will be from the point of randomisation until the 6-month follow-up. Intention-to-treat analysis will be applied.

The primary outcome for our economic evaluation is the Quality Adjusted Life Years (QALYs), to be converted from the CHU-9D data by applying for the UK tariff [47]. Secondary outcomes include the EQ-5D-Y-3L and the Delaware Class Climate score. The outcomes data will be collected at baseline, post-intervention, and 6 months follow-up using the e-CRFs.

Resource use includes three groups, i.e. (1) time spent by teachers/psychologists on preparing and delivering *Lina* and usual sessions as well as other professionals’ time spent on delivering *Lina* and usual sessions, (2) resource use for providing necessary equipment to deliver Lina sessions and (3) time spent by teachers/psychologists and other professionals on providing additional support to students as well as managing students’ disruptive behaviours. We will collect relevant unit cost data to convert the quantity of resource use for each item to monetary values. The cost for resource use will be presented at student and time point level (i.e. baseline, immediately after intervention and 6 months follow-up). We will explore the appropriate approach to handle missing values in costs and outcome data. No discounting will be required for costs and outcomes data.

A cost-utility analysis will be performed on each country’s data to estimate the incremental costs of *Lina* compared to usual lessons per additional QALY gained (or the Incremental Cost-Effectiveness Ratio (ICER)). We will report the uncertainties around the point estimate of the ICER. Also, the point estimate will be compared against the country-specific willingness-to-pay threshold(s). We will pool data from the three countries together to conduct a multiple-country cost-utility analysis. A few sensitivity analyses will be performed including (1) using the EQ-5D-Y-3L and the Delaware Class Climate score as outcome measures (2) conducting economic evaluation under alternative scenarios related to implementation (e.g. frequency of session delivery) to help contextualise the findings for future implementation (3) applying for alternative approach(es) to handle missing values.

A Health Economic Analysis Plan will be signed off before the start of the economic evaluation.

#### Qualitative analysis plan

We will adopt a critical realist epistemological approach to analysing qualitative data. Focus group discussions and individual interviews will be digitally recorded (with consent) and transcribed verbatim in each country’s language. Transcripts will be anonymised before analysis. We will conduct reflexive thematic analysis using a phased coding approach in NVivo 12 Plus.

In phase 1, a select team of researchers across all trial sites (UK, Czechia and Portugal), and a gender champion (trained in qualitative coding techniques) will collaboratively develop a coding framework. Each team will conduct open coding on 3–5 initial transcripts per site to generate a coding framework, reviewing codes collaboratively for coherence, consensus and relevance to primary research questions. This framework will guide further exploration within specific areas of inquiry (i.e. will be used to constrain the data set to address specific research questions). Following the development of the coding framework, we will iteratively develop a multi-lingual comprehensive codebook via local language open and focused coding.

In phase 2, each country team will then code all qualitative data using the agreed framework with discrepancies reviewed collaboratively and new codes approved by the coordinating team for framework consistency.

Regular fortnightly meetings will be held to with the research teams and gender champion to incorporate multiple perspectives during both phases to strengthen the coding outcomes for coherence, consensus and relevance to primary research questions. Results will be structured into thematic categories that highlight how the evidence addresses the primary questions on Lina experiences and how these interact with vulnerability using reflexive thematic analysis.

#### Implementation evaluation analysis plan

The implementation evaluation will follow a mixed-methods design triangulating quantitative and qualitative data to assess feasibility, acceptability, appropriateness and fidelity of Lina delivery across countries.

Quantitative data on acceptability, feasibility and appropriateness from the teacher implementation measures will be analysed descriptively, reporting means, standard deviation and proportions of teachers a range of responses for each measure.

Fidelity will be assessed from structured observation forms, calculating the percentage of core Lina components delivered per session and comparing fidelity rates across countries using descriptive statistics. Teacher focus group discussions will be analysed using deductive thematic analysis with a priori codes derived from the primary research questions.

Quantitative teacher scores will be triangulated with qualitative themes from focus groups and structured observation data to examine alignment between teacher-rated implementation experiences and observed fidelity. Through this, we hope to identify contextual factors (barriers and facilitators) influencing implementation across the trial sites. Findings will be organised by country context to examine site-specific variations in implementation.

### Who will be included in each analysis {27b}

All randomised clusters (i.e. classroom) and their enrolled participants will be included in the primary analyses according to the intention-to-treat (ITT) principle, whereby participants will be analysed in the group to which their classroom was originally allocated (i.e. control or intervention).

### How missing data will be handled in the analysis {27c}

Analysis will follow the intention-to-treat principle, meaning all participants will be analysed in the groups to which their classes were assigned, regardless of protocol deviations where contamination may have occurred. Any protocol deviation will be reported to the Trial Manager and formally documented.

The proportion of missing data from each instrument in the trial assessments (i.e. student and teacher CRFs) will be summarised across assessment timepoints and their reasons will be described and discussed keeping in mind contextual realities within each trial site.

Missing data will be handled by multiple imputation by chained equations using the MI package in STATA and efforts will be made to ensure that imputed values stay within the valid range of each questionnaire. Results from the imputed dataset will then be combined using Rubin’s rules to produce final estimates that account for uncertainty due to missing data.

### Methods for additional analyses (e.g. subgroup analyses) {27d}

Additional and subgroup analyses will be fully detailed in the statistical analysis plan, including sensitivity analyses to examine the impact of missing data imputation.

Subgroup analyses will explore differential treatment effects across genders and across high (top quartile) versus not high (lower three quartiles) baseline levels of social anxiety (SAS-A).

The effect of adherence on sense of belonging (DCCS) will be examined via comparison of participants who received “sufficient” *Lina* to those that did not receive *Lina* or did not complete “sufficient” *Lina. *“Sufficient” *Lina* is defined as at least 4 *Lina* sessions, including the last 2 and either the first or second (based on the content of each session in the context of current knowledge on mechanisms of change).

### Interim analyses {28b}

No interim analysis will be conducted. A trial steering committee composed of relevant cross-disciplinary professionals from the Czech Republic, Portugal and the UK (4 members), will have the power to terminate the trial based on regular reports on implementation, including adverse events.

### Protocol and statistical analysis plan {5}

A full statistical analysis plan (SAP) will be developed prior to exporting data from the database and reviewed by the trial steering committee. Data analysis will not be conducted prior to final sign off of the SAP. The SAP will be made publicly available prior to data lock. Once country level data has been analysed, then data from across the three countries will be pooled and analysed as one dataset. This will follow analysis as described above but with further level of clustering at the country level. The full analysis plan for the pooled analysis will also be described in the SAP. The trial statistical analysis plan (SAP) will be finalised before the database lock and published on University of Birmingham’s OSF record as well as to the study protocol as an update at a later stage. This study protocol will be made publicly accessible through publication in a peer-reviewed journal as well as the ISRCTN record (https://www.isrctn.com/ISRCTN11613092).

## Oversight and monitoring

### Composition of the coordinating centre and trial steering committee {3d}

The trial manager is based at Queen Mary University of London, where trial statistics and health economist teams are also based. These researchers will not play a direct role in data collection, but will form part of the Trial Management Group (TMG). The TMG will additionally comprise leads of the teams carrying out *Lina* implementation in each country, and at least one representative from each team actually carrying out implementation on the ground. The two design leads of *Lina* will also be part of the TMG. The TMG will meet weekly for the duration of the trial.

The trial steering committee (TSC) is independent of the TMG. It includes key professions relevant to the trial and its members are distributed among the 3 participating countries. A trialist and statistician is based in the UK and will independently review the SAP. An education practitioner with additional experience in research is based in the Czech Republic. A psychologist with experience in youth mental health is based in Portugal. Finally, a youth worker from the UK will represent both lay and youth voices.

The TSC will meet once before the trial commences and from then on, at 3–6-month intervals until data collection and analysis are complete. The committee will receive monthly reports on ongoing implementation, including any adverse events and will have the responsibility to call exceptional meetings should any concerns arise. Following their activity, they will produce a final report summarising their work and any lessons learned.

### Composition of the data monitoring committee, its role and reporting structure {28a}

We do not have a separate Data Monitoring Committee, but the Trial Steering Committee will also act as the Data Monitoring and Ethics Committee (DMEC) and will be responsible for the review of the safety reports, e.g. number of serious adverse events.

### Frequency and plans for auditing trial conduct {29}

Oversight will be maintained through regular (weekly) virtual check-ins and in-person site visits to the trial sites in the UK, Portugal and Czech Republic conducted by the Trial Manager (from Queen Mary University London) to ensure adherence to the protocol, data quality, trial procedures and documentation.

### Protocol amendments {31}

Any important protocol modifications (e.g. changes to eligibility criteria, outcomes, analysis plans, or trial procedures) will be communicated to and discussed with the Trial Management Group (includes the Chief Investigator, site lead, trial lead and manager, site researchers, statistician and health economist) and the Trial Steering Committee (TSC). Amendments will be updated in the trial registry (ISRCTN) and shared with trial researchers. All approved amendments will be documented with version control.

### Dissemination policy {8}

Trial findings will be disseminated following the ASP*belong* programme’s dissemination and communication plan, which is reviewed and revised regularly and comprises a deliverable of the project, publicly available via the European Commission. The plan includes a commitment to fully open access publications.

## Discussion

This trial has been designed to evaluate the effectiveness of *Lina*, a novel, game-based digital intervention intended to improve classroom-level belonging among children ages 12–13 across three European countries. As a cluster Randomised Controlled Trial with a pragmatic approach, the study design and procedures have been intentionally aligned within routine educational systems and structures to improve recruitment rates and operational feasibility.

In selecting the unit of randomisation, we weighed up the increased risk of contamination, which would come with class as the unit of randomisation, with the expectation that our recruitment and retention would be greatly facilitated by ensuring that all involved schools had some classes engaged with the intervention throughout. Particularly given the non-explicit nature of the *Lina* intervention, which aims to improve class climate and belonging by the specific experiences of collaboration acquired by experiencing *Lina* together, we judged the risk of contamination to be relatively small compared to that of the expected recruitment and retention challenges that would have come from using school as a unit of randomisation.

Despite the intention for trial to be as similar as possible across the three countries, local contexts have required substantial specific considerations. Some of these have been integrated into the overall design, and others have led to country-specific procedures. For example, the core requirement for prior local validation of any student-facing questionnaires used in Portugal was one of the primary criteria we considered in selection of our outcome measures, and doing this has allowed us to maintain consistency in effectiveness outcome measures across countries. However, the legal requirement in Portugal for any intervention in the realm of psychological wellbeing (i.e. that could be badged as psychotherapeutic) to be administered by psychologists has required a different plan for implementation in Portugal relative to the other sites. When judging where to make country-specific modifications and where to push for consistency, we considered the long-term picture of likely implementation “in the wild”. Thus, where country-specific context suggested that a particular scenario would likely be relevant to long-term use of *Lina* in that setting (e.g. the Portuguese example of needing psychologists to implement the programme), we were more inclined to make those country-specific modifications to trial design.

Blinding was an issue which we regularly and widely debated within our TMG. Given the nature of the intervention, neither students, teachers nor observing researchers can be blind to the intervention versus control group allocation. However, we had originally intended that researchers collecting data would be blind to the allocation. During the detailed process of laying down scheduling and teams in each implementation site however, it quickly became apparent how challenging it would be to keep any team members blind to allocation. We are aiming to achieve this where possible, but it is highly dependent on the practical set-up of each implementing team. And in the eventuality of any illness or other unexpected events within the teams, it is likely that at least some of the blinding will be jeopardised. We also considered maintaining the statistician conducting the analysis blind to allocation. However, in the context of the additional pragmatic challenges (resource demands) that would entail, we deemed that the prior full specification of the statistical analysis plan would appropriately minimise that risk of bias.

As the first evaluation of any Augmented Social Play intervention post early stage piloting of a *Lina*, proof-of-concept, these trials will be definitive in terms of assessing the strength and scope of the benefits that may be mediated by ASP. Although ASP, including *Lina*, represents an entirely novel combination of active elements, each of these (e.g. fictional story, collaborative puzzle solving, mobile-device mediated, evidence-based psychotherapeutic techniques) is well known within its primary disciplinary field. Results of these trials therefore have the potential to stimulate exciting new transdisciplinary work towards securing youth mental health and wellbeing, within a specific ASP context and beyond.

## Trial status

**Protocol version number:** v2.21

**Protocol Date:** 24 April 2026.

**Date recruitment started:** 22 September 2025.

**Approximate date recruitment will be complete:** 30 January 2026.

## Supplementary Information

Supplementary Material 1

Supplementary Material 2

## Data Availability

Data will be made open access by the time of publication of corresponding results at the latest.

## Abbreviations

AE: Adverse events
ASP: Augmented Social Play
CRFs: Case report forms
CZ: Czechia
DMEC: Data Monitoring and Ethics Committee
eCRFs: Electronic case report forms
GDPR: General Data Protection Regulation
ICER: Incremental cost-effectiveness ratio
ICC: Intraclass correlation coefficient
PID: Participant ID
PSHE: Personal, Social, Health and Economic education
PT: Portugal
QALYs: Quality-adjusted life years
REC: Research Ethics Committee
SAE: Serious adverse event
SEND: Special educational needs and disabilities
SAP: Statistical analysis plan
TMF: Trial Master File
TMG: Trial Management Group
TSC: Trial Steering Committee
UK: United Kingdom
