# Supplementary material for: Three cluster randomised controlled trials in Portugal, Czechia and the United Kingdom to test the effectiveness and cost-effectiveness of an augmented social play intervention in schools to support real-world connection and sense of belonging (Lina trial protocol)
Source: Trials. 2026 Jun 3;27:516. doi: 10.1186/s13063-026-09780-7 (PMC13445914; doi:10.1186/s13063-026-09780-7)
Supplement: Supplementary file 1 [file 13063_2026_9780_MOESM1_ESM.docx]

**Appendix 1 – Information Sheets and Consent Forms**

**1. ORGANISATIONAL AGREEMENT FOR SCHOOLS**

**Organisational Information Document**

***Lina***

**Study Director: Dr. Kate Anne Woodcock**

You are receiving this document as your school has indicated an interest in participating in *Lina*. If you have any further questions, please do not hesitate to reach out to the research team using the contact details given at the end of this document.

**Project Overview**

As you know, *Lina* is an interactive classroom program where young people use smartphones provided by the research team to engage in a fictional mystery and solve puzzles together. Through collaborative activities, *Lina* promotes essential skills such as teamwork, communication, and problem-solving, with the aim of strengthening students’ sense of belonging and overall wellbeing in the classroom in an enjoyable and educational way.

In this research, we want to evaluate *Lina* to determine scientifically how and in which ways it strengthens students’ sense of belonging and wellbeing - this includes looking at students’ ratings of key things we are aiming to influence and also talking to some students in more depth about their experiences. We also want to get a full understanding of what it costs to do *Lina*. Finally, we want to use your feedback to continue to develop the way we support schools to do *Lina*.

This project has ethical approval from the University of Birmingham’s STEM Research Ethics Committee.

**What does participating in the trial for *Lina* entail for schools?**

- All schools choosing to participate in the trial will be replacing some usual school lessons with *Lina* and schools will not deem this to be outside of the scope of usual school activities. Therefore, participating in *Lina* does not require parental consent.
- In this research, we will compare classes that are doing *Lina* with those that are not doing *Lina* now (but will do it later). This means that around half of the classes in your school doing *Lina* will do so at the beginning of the 2025/26 academic year (when they are in Year 8) and the other half will do so at the beginning of the 2026/27 academic year (when they are in Year 9). We need to determine randomly which classes do *Lina* when, and must do this after all classes have completed our initial assessment session (in September 2025).
- All involved classes will participate in 3 data collection points - September 2025, before Christmas 2025 (after the classes doing *Lina* have finished), and around June 2026 (6 months later).
- Researchers will collect different types of data to evaluate the effectiveness and impact of *Lina* on young people’s wellbeing in the classroom. This will include:
  1. Questionnaires with young people and teachers
  2. Focus group discussions with young people and teachers
  3. Interviews with young people
  4. Structured observations of *Lina* being implemented in classrooms
- Young people and parents/guardians will have the right to withdraw from participating in any data collection activities at any point. However, they will still receive *Lina* as part of standard school lessons.
- Teachers will also have the right to withdraw from data collection activities, but if the school does not wish to withdraw the teacher’s class from the trial, they would need to arrange a replacement teacher.
- All collected data will be confidential and stored securely at the University of Birmingham. We will use the information we collect to write reports, which we will publish in journals and talk about at conferences. None of these reports will contain any names.
- We may also share information we collect with other researchers in the future. This information would not contain any names.

**What will happen after the trial is completed?**

We will be keeping you informed about the findings of the study as and when they are published.

We will also share any information on any further developments to *Lina*.

**Contact Details:**

If you have any questions, or if you would like regular detailed updates at each step of the study, then please feel free to contact the research team as follows:

**Tamanna Malhotra: txm390@bham.ac.uk**

**Alison Fulop: axf551@bham.ac.uk**

Kate Woodcock (study director): k.a.woodcock@bham.ac.uk

Phone Number: 0121 414 6036

Please sign and date the form below to indicate that you have understood what participation in *Lina* entails, and that you are happy for your school to participate.

School Name:	______________________

Staff Name:	______________________

Signature:	______________________

Date:	______________________

**2. SCHOOL AND TEACHER INFORMATION SHEET**

**School Information Sheet for Evaluating Lina**

**Study Director: Dr. Kate Anne Woodcock**

You are receiving this information sheet as your school has chosen to implement Lina within the classroom curriculum to enhance the student experience.

The research is sponsored by the University of Birmingham, and is led by a large team of people from different countries and backgrounds, including Dr. Kate Anne Woodcock, who is the scientific coordinator of the study.

For any questions or concerns, you can contact the research team at the contact details given at the end of this document.

**Project Overview and Aims**

As you know, Lina is an interactive classroom program where young people use smartphones provided by the research team to engage in a fictional mystery and solve puzzles together. Through collaborative activities, Lina promotes essential skills such as teamwork, communication, and problem-solving, with the aim of strengthening students’ sense of belonging and overall wellbeing in the classroom in an enjoyable and educational way.

In this research, we want to evaluate Lina to determine scientifically how and in which ways it strengthens students’ sense of belonging and wellbeing - this includes looking at students’ ratings of key things we are aiming to influence and also talking to some students in more depth about their experiences. We also want to get a full understanding of what it costs to do Lina. Finally, we want to use your feedback to continue to develop the way we support schools to do Lina.

This project has ethical approval from the University of Birmingham’s STEM Research Ethics Committee.

**Structure of Participation**

An important necessary part of scientific evaluation is that we need to compare what we are evaluating to something else. In this research, we will compare classes that are doing Lina with those that are not doing Lina now (but will do it later). This means that around half of the classes in your school doing Lina will do so at the beginning of the 2025/26 academic year (when they are in Year 8) and the other half will do so at the beginning of the 2026/27 academic year (when they are in Year 9). We need to determine randomly which classes do Lina when, and must do this after all classes have completed our initial assessment session (in September 2025).

The research activities fit around classes’ involvement in Lina and include:

1. All involved classes will participate in 3 data collection points - September 2025, before Christmas 2025 (after the classes doing Lina have finished), and around June 2026 (6 months later).
2. Discussions with students/ teachers between January and June 2026, each lasting no more than 1 hour, including:
   a) one-to-one discussions with a few self-selecting students who have done Lina
   b) b) 1 or 2 group discussions with a maximum of 6 students who have done Lina in each group
   c) a group discussion with teachers who have done Lina
3. Teachers of classes doing Lina from September 2026
   a) completing some brief questions (no more than 20 minutes) after each Lina lesson; and after the class has finished Lina
   b) taking part in a group discussion (no more than 1 hour) after their class has finished doing Lina

- **Location:** The study will take place at young people’s schools.
- **Our Involvement:** Members of our project team, including Tamanna Malhotra and Alison Fulop, will be on-site to facilitate sessions, assist with technical support, and guide the activities in a way that minimises disruption to school routines. Other members of the research team may also be present.

**What Will Young People Do?**

Participation is voluntary. Researchers will collect insights from young people through questionnaires, which will cover wellbeing and mental health, experiences of stigma, social skills, peer relationships, and feelings of classroom belonging. These assessments will help researchers understand the programme’s impact and what young people need to feel a stronger sense of belonging.

**What will teachers do?**

Participation is voluntary. Researchers will use questionnaires and focus groups to gather insights on your experience of delivering Lina.

The activities will include:

- Completing questionnaires to give feedback on your experience of implementing Lina. • Answering verbal questions about what went well/did not go well, and what improvements can be made.
- Participating in group discussions to give detailed feedback on implementing Lina.

Researchers will also observe classroom interactions between students and teachers during Lina. Observations will focus on aspects such as engagement levels, enthusiasm, time taken to complete activities, and group cohesion.

**Benefits to your School Community**

By participating, your school contributes to developing a resource that could play a significant role in supporting student well-being. Our team will be available to share findings and insights with your staff, enhancing their understanding of fostering belonging in the school environment. Your school will be part of an international network of participating schools in the UK and the EU, and we will endeavour to facilitate networking and collaboration.

**Risks and Safeguarding**

Some of the questionnaires in this study relate to belonging; and social and emotional wellbeing, which could evoke challenging emotions. While we’ve worked carefully with young people to ensure these questions are as sensitive as possible, we acknowledge that some topics might feel difficult for some individuals.

Safety and wellbeing will always be held as a greater priority than the aims of the study. All participants are free to take a break, or stop participating at any point without giving any reason. We have a dedicated safeguarding policy to ensure that all activities are conducted in a way that feels safe and comfortable.

This project has received ethical approval from the Science, Technology, Engineering and Mathematics Research Ethics Committee at the University of Birmingham. As part of the ethical approval process, we have also completed a risk assessment to ensure that young people’s safety and wellbeing is always prioritised.

**Privacy**

We will not disclose which young people and teachers participate to anyone outside of the research team. All information will be confidential, so will not include participants’ names. The research team will only keep a record of enough information about participants’ names to allow all of the research activities to be completed. After this, there will be no link between names and other information collected.

It will not be possible to keep anything shared within a group discussion private from other members of the group. Members of group discussion will be reminded about this at the beginning of the discussion.

If a participant does or says something that makes us think that they are in danger, or someone else is in danger, then we are legally obligated to report this information to appropriate safeguarding services.

**Consent**

We will fully inform parents and young people about what participation entails. We will work with you to ensure we use the best means in the context of your school to let parents and students know about the forthcoming research in summer 2025, before giving all of the information again in September 2025.

For students completing the questionnaires, we will ask parents to opt their child out of the research if they wish to before the first data collection lesson. At the beginning of the first data collection lesson, we will ask young people to document that they have understood what participation entails, had the opportunity to ask questions and agree to take part using yes/no questions. Only those students agreeing to take part will go on to complete the questionnaires.

For students self-selecting to additionally take part in one-to-one / group discussions, we will ask parents and students to complete an additional written consent form, or send us a consent email.

We will ask teachers to complete a consent form (or send us an email) to document that they understand what participation entails, and that they consent to participate. This form or email will contain the teacher’s name and contact details, and will be stored securely.

**Withdrawal**

Before young people have finished completing questionnaires either they or their parents can withdraw from the research at any point by letting the research team know. This can be via the researcher contact details on the information sheet or by the student letting a researcher know during a data collection session. However, any questionnaire data already collected from young people cannot be destroyed.

For students self-selecting to additionally take part in one-to-one / group discussions, they or their parents can withdraw in the same way at any point up to and during the data collection session. However, the only information we could destroy if requested is that collected from a one-to-one discussion, and young people /parents have 2 weeks after the discussion to request

this.

Teachers may withdraw at any point by contacting the research team (contact details are given at the end of this information sheet). However, if a teacher withdraws and the school would like their class to continue in the evaluation, it will be necessary for the school leadership team to identify a replacement teacher who consents to take part. It will not be possible for us to destroy any data already collected from teachers who withdraw.

Anyone choosing to withdraw will not be asked to give a reason and it will not affect their access to Lina or any other resources.

**Recording Information and Data Security**

1. **Participant Information and Consent**

We will record minimal information on participants’ names/contact details for us to be able to complete our research activities. These details will be stored on a secured database software hosted at the University of Birmingham.

2. **Questionnaires**

Responses will be collected and stored on a secured database software hosted at the University of Birmingham. All responses will be anonymised, and the database will only be accessed by primary researchers.

3. **Focus Group Discussions**

Focus groups and interviews will be audio recorded. Audio recordings will be stored on a secured database software hosted at the University of Birmingham. We will also write down what was discussed in focus groups, however, these notes will be completely anonymous and unidentifiable.

4. **Observations**

Observations will be recorded on an observation sheet, without reference to individuals or names. These sheets will be stored securely at the University of Birmingham.

All data is stored and processed in accordance with General Data Protection Regulation (GDPR) guidelines.

**What Happens Afterwards?**

We will use the information we collect to write reports, which we will publish in journals and talk about at conferences. None of these reports will contain any names. We may share information we collect with other researchers in the future. This information would not contain any names.

We will make sure that schools and teachers stay up to date with any developments of Lina. If young people, parents/guardians, and teachers would like an update about our research afterwards, they may contact us using the details at the end of this sheet.

**Questions or Concerns**

If you have any questions about this information, please contact the research team as follows: **Tamanna Malhotra: txm390@bham.ac.uk**

**Alison Fulop: axf551@bham.ac.uk**

Kate Woodcock (study director): k.a.woodcock@bham.ac.uk

Phone Number: 0121 414 6036

If you have a problem that the research team cannot solve, you can contact the Research Governance Team at the University of Birmingham on

researchgovernance@contacts.bham.ac.uk.

**3. TEACHER CONSENT FORM**

**Evaluating *Lina***

**Teacher Consent Form**

**Study Director: Dr. Kate Anne Woodcock**

Thank you for considering being part of *Lina*, which explores how to improve students’ sense of belonging and connectedness in the classroom. Please read the statements below and confirm your agreement by ticking the boxes provided.

In case of any questions, please reach out to a member of the research team using the contact details at the end of this document.

|  |  | **Teacher** |
| --- | --- | --- |
| 1 | I have read the evaluating *Lina* information sheet and know what participation will entail. |  |
| 2 | I understand that a researcher will be present during sessions to observe *Lina* in classrooms. |  |
| 3 | I know that the team might share results from the research in reports, conferences, etc. but this will not contain any names or identifiable information. |  |
| 4 | I know that the team might share information with other researchers in the future, but this will not contain any names or identifiable information. |  |
| 5 | I agree to participate in questionnaires to inform researchers about my experience of delivering *Lina*. |  |
| 6 | I agree to answer questions about what went well and what needed improving. |  |
| 7 | I agree to participate in a group discussion, providing more detailed feedback on my experience of implementing *Lina*. |  |

**Please fill in your details below:**

Name of School: Teacher’s name:

Contact (Phone number or Email address): Signature:

Date:

**If you would prefer to send us an email instead of filling in the form, please send us a copy of the email below (Tamanna Malhotra: txm390@bham.ac.uk) and write your name in the place indicated in red:**

*My name is [insert your name].*

*I give my permission to participate in Lina and support its implementation in my classroom.*

*________________________________________________________________________________*

If you have any questions or change your mind, you can talk to your teacher or contact the research team as follows:

**Tamanna Malhotra: txm390@bham.ac.uk**

**Alison Fulop: axf551@bham.ac.uk**

Kate Woodcock (study director): k.a.woodcock@bham.ac.uk

Thank you for thinking about joining *Lina*!

**4. PARENT INFORMATION SHEET**

**Parent Information Sheet for *Evaluating* *Lina***

**Study Director: Dr. Kate Anne Woodcock**

You are receiving this information sheet as your child’s school has chosen to implement *Lina* within their classroom curriculum to enhance the student experience.

We are therefore inviting the young person in your care to take part in *Evaluating* *Lina*. The research is sponsored by the University of Birmingham, and is led by a large team of people from different countries and backgrounds, including Dr. Kate Anne Woodcock, who is the scientific coordinator of the study.

For any questions or concerns, you can contact the research team using the contact details given at the end of this document.

**What are the aims of the research project?**

*Lina* is an interactive classroom program where young people use smartphones provided by the research team to engage in a fictional mystery and solve puzzles together. By collaborating with peers, they develop essential skills such as teamwork, communication, and problem-solving in an enjoyable and educational way.

A key focus of Lina is the time students spend face-to-face working together. The program is designed to create shared experiences that foster teamwork and meaningful connections within the class. This project has ethical approval from the University of Birmingham’s STEM Research Ethics Committee.

**Where will the work take place?**

The study will take place at young people’s schools.

**What will young people do?**

Participation in this evaluation research is voluntary.

Your child will be doing *Lina* with their class starting from either September 2025 or September 2026. If they do it first, a member of the development team will be there to observe how it goes and take structured notes. These will not contain any details about individuals.

Young people will be asked to complete questionnaires on their phones, covering topics such as mental health and wellbeing; and relationships with peers.

Questionnaires will be completed three times - in September 2025, before Christmas 2025 and around June 2025.

This will help researchers understand the impact of *Lina* on young people’s wellbeing within the classroom.

We will speak to some young people about their experiences of *Lina* in more detail (either 1:1 or in a small group with other young people). We will put up a poster in young people’s classrooms telling them how to volunteer. Teachers may also identify particular young people for us to approach. We need to speak to a range of young people with one of a number of experiences (e.g. good or bad previous experiences with peers, good or bad experience of *Lina*). Any young person who volunteers or is identified by their teacher will receive additional information about this part of the research, as will their parents. If they wish to take part in the 1:1 or group discussion, the young person and their parents will then have to give us their permission.

**Who will be involved in the activities young people do?**
Members of our project team will be involved, including Tamanna Malhotra and Alison Fulop, who are doctoral researchers working at the University of Birmingham. Other members of the research team may also be present.

**Could taking part lead to anything good happening?**
Participating in the study allows young people to actively shape the development of an innovative large-scale international programme that aims to benefit young people’s wellbeing.

**Could taking part lead to anything bad happening?**
Some of the questionnaires in this study relate to social and emotional wellbeing and may prompt young people to think about challenges they might face at school or with their peers. While we’ve worked carefully with young people to ensure these questions are as sensitive as possible, we acknowledge that some topics might feel difficult for some individuals.

The safety and wellbeing of all young people is our highest priority. Participation is entirely voluntary, and young people can take a break or stop participating at any time without giving a reason.

**Privacy**

We will not tell anyone outside of our team which young people take part. Any information we record on young people’s names will only be to the extent that is strictly necessary to complete our research. The answers young people give to the questionnaires will not be stored in the same place as any information on their name.

If a young person does or says something in a questionnaire or observation that makes us think that they are in danger, or someone else is in danger, then we are legally obligated to report this information to appropriate safeguarding services.

**Consent**

If when reading this information, you or your child decide that you do not want your child to take part and complete the questionnaires, please let us know by (insert date). Before completing the questionnaires for the first time, your child will be asked to confirm whether they agree to take part. They will only complete the questionnaires if they agree to do so.

**Withdrawal**

Even if your child agrees to complete the questionnaires, they can change their mind at any time and without giving a reason, up until the final questionnaire is complete. You can also withdraw your consent by contacting the research team (**kwrg@contacts.bham.ac.uk**). If your child withdraws, any responses to questionnaires that your child has already given will already be part of our research, so we cannot destroy them.

**What data will be collected and how is it stored?**

Young people’s responses to questionnaires will be stored as numbers on a secured database software hosted at the University of Birmingham. Responses will be stored separately from any information on young people’s names and will be made anonymous after data collection is complete (no link to names). All data is stored and processed in accordance with General Data Protection Regulation (GDPR) guidelines.

**Compensation**

Young people will not receive any compensation for taking part.

**What will happen afterwards?**

We will use the information we collect to write reports, which we will publish in journals and talk about at conferences. None of these reports will contain any names. We may share information we collect with other researchers in the future. This information would not contain any names.

We will make sure that young people’s schools and teachers stay up to date with any developments of *Lina*. If you would like an update about our research afterwards, please contact us using the details at the end of this sheet.

**Researchers contact details:**

If you have any questions about this information, please contact the research team as follows:

**Tamanna Malhotra:** [**txm390@bham.ac.uk**](mailto:txm390@bham.ac.uk)

**Alison Fulop:** [**axf551@bham.ac.uk**](mailto:axf551@bham.ac.uk)
Kate Woodcock (study director): [k.a.woodcock@bham.ac.uk](mailto:k.a.woodcock@bham.ac.uk)

Phone Number: 0121 414 6036

**To withdraw your child, please email the team at kwrg@contacts.bham.ac.uk**

If you have a problem that the research team cannot solve, you can contact the Research Governance Team at the University of Birmingham: [researchgovernance@contacts.bham.ac.uk](mailto:researchgovernance@contacts.bham.ac.uk)

**5. YOUNG PEOPLE INFORMATION SHEET**

**6. SCRIPT TO INFORM YOUNG PEOPLE IN CLASSROOMS**

*The following script is to be read aloud to all young people taking part in the Lina trial before baseline data collection.*

Researchers contact details for reference**: Tamanna Malhotra:** [**txm390@bham.ac.uk**](mailto:txm390@bham.ac.uk); **Alison Fulop:** [**axf551@bham.ac.uk**](mailto:axf551@bham.ac.uk); Kate Woodcock (study director): [k.a.woodcock@bham.ac.uk](mailto:k.a.woodcock@bham.ac.uk); Phone Number: 0121 414 6036

Dear pupils at (insert school name),

Your class has been chosen by researchers at the University of Birmingham to try out an exciting new activity called *Lina*. Some classes will do *Lina* starting in September 2025 and some will do *Lina* starting in September 2026.

**What is *Lina*?**

We don’t want to spoil the surprise about what you will be doing, but we can say you will be playing *Lina* with your classmates and solving a mystery. You will have to wait until your first *Lina* lesson to find out more!

**What is the research?**

- We invite you to take part in research answering questions about your experience and how it made you feel. The questionnaires are about mental health and wellbeing, and interacting with your peers. Even the classes that don’t start playing Lina until September 2026 will be asked to fill out theses questionnaires this year
- We'll ask you to complete the questionnaires 3 times - at the beginning of the school year, before Christmas and towards the end of the year

**Important points**

- There are no right or wrong answers
- We will look at the answers to the questionnaires to find out whether, on average, doing *Lina* changes anything about young people's mental health or wellbeing. We won't be using your name or anything that identifies you as an individual in our analyses.
- You do not have to take part in questionnaires. You can ask to take a break or stop at any point.
- We will not use your name or any other identifiable information about you when we tell people what we find out
- We will have to tell someone if we think you or someone else are in danger.
- We will share the information we collect in reports and at conferences, but no information will contain any names, and will be completely anonymous.

**If you change your mind**

- You can change your mind and stop participating in the questionnaires at any point by speaking to (or contacting) the researchers or telling your teacher
- If you do this, we are unable to remove any answers you have already given from our study, and you will still play Lina as part of regular school lessons.

**Where can I get more information?**

If you would like to contact the researchers to ask any questions, their details will be on the information you will be given, or you can ask me (or your teacher).

**7. YOUNG PEOPLE DEBRIEF SHEET**

**Debrief Letter**

**Evaluating *Lina***

**Study Director: Dr. Kate Anne Woodcock**

Thank you for your participation in evaluating *Lina*.

Your participation will inform the development and wider-scale implementation of *Lina*. We will be using the collected data to better understand the ways in which *Lina* increases belongingness and wellbeing for young people in schools.

We will be keeping you informed about the findings of the study as and when they are published. We will also share any information on any further developments to *Lina*.

If you have any questions, or if you would like regular detailed updates at each step of the study, then please feel free to contact the research team as follows:

**Tamanna Malhotra: txm390@bham.ac.uk**

**Alison Fulop: axf551@bham.ac.uk**

Kate Woodcock (study director): k.a.woodcock@bham.ac.uk

Phone Number: 0121 414 6036

If you feel like you need some support with any negative feelings, please reach out to:

(insert school specific wellbeing resources)

**Information regarding mental health and wellbeing resources is given below (to be adapted depending on country):**

● If you're feeling overwhelmed and like you can't cope, you can:

(A) Call HOPELINEUK on 0800 068 4141 or the Samaritans on 116 123.

(B) Text SHOUT to Shout's textline on 85258.

(C) Call the NHS on 111 and select option 2.

● For a longer list of helplines and organisations for young people, please visit - https://www.mind.org.uk/for-young-people/how-to-get-help-and-support/useful-contact s/

**8. RECRUITMENT POSTER FOR YOUNG PEOPLE FOCUS GROUPS**

**9. RECRUITMENT POSTER FOR YOUNG PEOPLE INTERVIEWS**

**10. PARENT INFORMATION SHEET FOR FOCUS GROUPS**

**Parent/Guardian Information Sheet for *Discussions about Lina - Focus Groups***

**Study Director: Dr. Kate Anne Woodcock**

You are receiving this information sheet as your child’s school has chosen to implement *Lina* within their classroom curriculum to enhance the student experience. We are therefore inviting the young person in your care to take part in *Discussions about Lina*. The research is sponsored by the University of Birmingham, and is led by a large team of people from different countries and backgrounds, including Dr. Kate Anne Woodcock, who is the scientific coordinator of the study.

For any questions or concerns, you can contact the research team using the contact details given at the end of this document.

**What are the aims of the research project?**

*Lina* is an interactive classroom program where young people use smartphones provided by the research team to engage in a fictional mystery and solve puzzles together. By collaborating with peers, they develop essential skills such as teamwork, communication, and problem-solving in an enjoyable and educational way.

A key focus of *Lina* is the time students spend face-to-face working together. The program is designed to create shared experiences that foster teamwork and meaningful connections within the class.

As a research team dedicated towards the improvement of youth wellbeing, we recognise the importance of highlighting young people’s voices in all aspects of research. We have worked closely with class teachers who have suggested that the young person in your care is likely to have an interesting and useful perspective about taking part in *Lina*. This is because your young person has a strong opinion about *Lina* (e.g. they thought *Lina* was really good or really bad). This perspective will contribute to the further development of *Lina*.

This project has ethical approval from the University of Birmingham’s STEM Research Ethics Committee.

**Where will the study take place?**

The study will take place at young people’s schools.

**What will young people be asked to do?**

Participation is voluntary. Young people are invited to participate in group discussions (sometimes called focus groups) with 3-5 other classmates and a researcher.

The focus groups will include activities such as:

- Giving feedback about playing *Lina* in class and talking about how we can make it better.
- Giving feedback on their general experience of playing *Lina*.
- Talking about feeling a sense of belonging or not feeling a sense of belonging.

We will agree on timings with the young people taking part so that it fits in with them and their school schedule.

**What data will be collected from young people and how is it stored?**

1. **Name and contact details**

We will ask parents/guardians and young people to complete a consent form (or send us an email) to document that they consent to participate in the research study, have had the opportunity to ask questions, and understand what participation involves. This form or email will contain the young person’s name and their parent/guardian’s name.

A copy of this form or email will be stored securely at the University of Birmingham.

2. **Focus groups**

Focus groups will be audio recorded. Audio recordings will be stored securely at the University.

All data is stored and processed in accordance with General Data Protection Regulation (GDPR) guidelines.

**Who will be involved in the activities young people do?**

Members of our project team will be involved, including Tamanna Malhotra and Alison Fulop, who are doctoral researchers working at the University of Birmingham. Other members of the research team may also be present.

**Could taking part lead to anything good happening?**

Participating in the study allows young people to actively shape the development of an innovative large-scale international programme that aims to benefit young people’s wellbeing. Being involved in the focus group will give young people an opportunity to express their opinions about participating in *Lina*. They will also have the opportunity to share their ideas on how we can improve the programme*.*

**Could taking part lead to anything bad happening?**

We might sometimes talk to young people about things that could make them feel like they don’t belong. We might also talk to young people about difficult experiences that might affect their belonging or difficult experiences that fictional characters might have.

Some of these topics might be difficult for some young people. However, young people’s safety and wellbeing will always be held as a greater priority than the aims of the study. All young people are free to take a break, or stop participating at any point without giving any reason.

**Privacy**

We will not tell anyone outside of our team which young people take part.

Young people who take part in the focus groups will join in with other young people. Everyone in the focus group will hear what people have to say. We will ask young people to follow “rules of the room” and not talk about what anyone has done or said after the focus group.

We will make a transcript of the audio recording of the focus group as soon as possible after the group. We will make sure that any information that might identify your child is removed from the transcript. We will then delete the recording.

If a young person does or says something that makes us think that they are in danger, or someone else is in danger, then we are legally obligated to report this information to appropriate safeguarding services.

**Consent**

If a young person wants to take part, we need them to give us their permission in writing. We also need their parent/legal guardian to give us their permission in writing. The young person and parent/guardian can complete our consent form or send us an email using the guidance on the attached consent form.

**Withdrawal**

If a young person changes their mind about taking part, they can stop participating in the focus group at any time. They will not be asked to share why they would like to stop taking part, and there will be no consequences of withdrawing from the study.

We won’t be able to remove any data that we have already recorded from the young person because it will include contributions from other young people who are still taking part.

**Compensation**

Young people will receive a certificate of participation.

**What will happen afterwards?**

We will use the information we collect to write reports, which we will publish in journals and talk about at conferences. None of these reports will contain any names. We may share information we collect with other researchers in the future. This information would not contain any names.

We will make sure that young people’s schools and teachers stay up to date with any developments of *Lina*. If you would like an update about our research afterwards, please contact us using the details at the end of this sheet.

**Questions or concerns**

If you have any questions about this information, please contact the research team as follows:

**Tamanna Malhotra: txm390@bham.ac.uk**

**Alison Fulop: axf551@bham.ac.uk**

Kate Woodcock (study director): k.a.woodcock@bham.ac.uk

Phone Number: 0121 414 6036

If you have a problem that the research team cannot solve, you can contact the Research Governance Team at the University of Birmingham on

researchgovernance@contacts.bham.ac.uk.

**11. PARENT INFORMATION SHEET FOR INTERVIEWS**

**Parent/Guardian Information Sheet for**

***Discussions about Lina – Interviews***

**Study Director: Dr. Kate Anne Woodcock**

You are receiving this information sheet as your child’s school has chosen to implement *Lina* within their classroom curriculum to enhance the student experience. We are therefore inviting the young person in your care to take part in *Discussions about Lina*. The research is sponsored by the University of Birmingham, and is led by a large team of people from different countries and backgrounds, including Dr. Kate Anne Woodcock, who is the scientific coordinator of the study.

For any questions or concerns, you can contact the research team using the contact details given at the end of this document.

**What are the aims of the research project?**

*Lina* is an interactive classroom program where young people use smartphones provided by the research team to engage in a fictional mystery and solve puzzles together. By collaborating with peers, they develop essential skills such as teamwork, communication, and problem-solving in an enjoyable and educational way.

A key focus of *Lina* is the time students spend face-to-face working together. The program is designed to create shared experiences that foster teamwork and meaningful connections within the class.

As a research team dedicated towards the improvement of youth wellbeing, we recognise the importance of highlighting young people’s voices in all aspects of research. We have worked closely with class teachers who have suggested that the young person in your care is likely to have an interesting and useful perspective about taking part in *Lina*. This is because your child receives Special Educational Needs support (including Gifted and More Able), or the school receives pupil premium on behalf of your child, or your child has a notable social experience (e.g. being part of a social extracurricular club; has many friends outside of school; has experienced or witnessed bullying in the past). This perspective will contribute to the refinement of *Lina* to make it as efficient as possible for other young people in the future.

This project has ethical approval from the University of Birmingham’s STEM Research Ethics Committee.

**Where will the study take place?**

The study will take place at young people’s schools.

**What will young people be asked to do?**

Participation is voluntary. Young people are invited to participate in a 1:1 discussion with a researcher (sometimes called an interview).

The interview will include activities such as:

- Talking about ideas, personal experiences, and identity
- Talking about feeling a sense of belonging or not feeling a sense of belonging
- Stories, drawings, or role-playing activities related to their experience of participating in *Lina*.

We will agree on timings with the young people taking part so that it fits in with them and their school schedule.

**What data will be collected from young people and how is it stored?**

1. **Name and contact details**

We will ask parents/guardians and young people to complete a consent form (or send us an email) to document that they consent to participate in the research study, have had the opportunity to ask questions, and understand what participation involves. This form or email will contain the young person’s name and/or their parent/guardian’s name. A copy of this form or email will be stored securely at the University of Birmingham.

2. **Interview**

The interviews will be audio recorded. Audio recordings will be stored on the University of Birmingham Research Data Store, which is a secure, password-protected research data repository hosted at the University of Birmingham. Access will be granted only to the primary research team, including the scientific coordinator.

We will also write down what young people tell us in the interview, however, these notes will be completely anonymous and unidentifiable.

All data is stored and processed in accordance with General Data Protection Regulation (GDPR) guidelines.

**Who will be involved in the activities young people do?**

Members of our project team will be involved, including Tamanna Malhotra and Alison Fulop, who are doctoral researchers working at the University of Birmingham. Other members of the research team may also be present.

**Could taking part lead to anything good happening?**

Participating in the study allows young people to actively shape the development of an innovative large-scale international programme that aims to benefit young people’s wellbeing. The interview will give young people an opportunity to express their opinions and thoughts, and share feedback with us that will significantly influence the refinement of *Lina*. This will help us make *Lina* as effective as possible when implemented with other young people in the future.

**Could taking part lead to anything bad happening?**

We might sometimes talk to young people about things that could make them feel like they don’t belong. We might also talk to young people about difficult experiences that might affect their belonging or difficult experiences that fictional characters might have.

Some of these topics might be difficult for some young people. However, young people’s safety and wellbeing will always be held as a greater priority than the aims of the study. All young people are free to take a break, or stop participating at any point without giving any reason.

**Privacy**

We will not tell anyone outside of our team which young people take part. The conversation will be 1:1 with a researcher unless your child asks for a trusted adult to be present.

We will record the conversation and transcribe it. Once we have a written transcript, we will destroy the recording. The transcript will not include your child’s name, and any information that could allow someone reading the transcript to identify your child will also be removed.

If a young person does or says something in an interview that makes us think that they are in danger, or someone else is in danger, then we are legally obligated to report this information to appropriate safeguarding services.

**Consent**

If a young person wants to take part, we need them to give us their permission in writing. We also need their parent/legal guardian to give us their permission in writing. The young person and parent/guardian can complete our consent form or send us an email using the guidance on the attached consent form.

**Withdrawal**

If a young person changes their mind about taking part, they can stop participating in the interview at any time. They will not be asked to share why they would like to stop taking part, and there will be no consequences of withdrawing from the study.

Young people will be able to tell us if they want us to remove the information they have given us. They will need to tell us within 2 weeks of taking part in the interview either by telling their teacher or by contacting us directly.

**Compensation**

Young people will receive a certificate of participation.

**What will happen afterwards?**

We will use the information we collect to write reports, which we will publish in journals and talk about at conferences. None of these reports will contain any names. We may share information we collect with other researchers in the future. This information would not contain any names.

We will make sure that young people’s schools and teachers stay up to date with any developments of *Lina*. If you would like an update about our research afterwards, please contact us using the details at the end of this sheet.

**Questions or concerns:**

If you have any questions about this information, please contact the research team as follows:

**Tamanna Malhotra:** [**txm390@bham.ac.uk**](mailto:txm390@bham.ac.uk)

**Alison Fulop: axf551@bham.ac.uk**

Kate Woodcock (study director): k.a.woodcock@bham.ac.uk

Phone Number: 0121 414 6036

If you have a problem that the research team cannot solve, you can contact the Research Governance Team at the University of Birmingham on

researchgovernance@contacts.bham.ac.uk

**12. YOUNG PERSON INFORMATION SHEET FOR QUALITATIVE EVALUATION**

**13. YOUNG PERSON AND PARENT CONSENT/ASSENT FORM**

**Discussions about *Lina***

**Young Person/Parent Consent Form**

**Study Director: Dr. Kate Anne Woodcock**

This is a consent form for young people participating in *Discussions about Lina*. Please ensure that you have read the attached information sheet before completing this agreement form. You may wish to speak to your parent or guardian for help with understanding this document.

If you are a young person, please enter your initials under the “Young Person” column. If you are a parent/guardian, please put your initials under the “Parent/Guardian” column. You can also reach out to a member of the research team using the contact details provided at the end of this document.

|  |  | **Young**  **Person initials** | **Parent/**  **Guardian**  **initials** |
| --- | --- | --- | --- |
| 1 | I have read the *Discussions about Lina* information sheet. |  |  |
| 2 | I know that young people are free to stop taking part at any time. |  |  |
| 3 | I know that if a young person stops taking part in the focus groups, the team will not be able to remove any information the young person has already given them. **(Not applicable if not taking part in focus groups)** |  |  |
| 4 | I know that all young people taking part in the focus group will know what others have said. **(Not applicable if not taking part in focus groups)** |  |  |
| 5 | I know I have two weeks to ask for my data to be withdrawn from the interview. **(Not applicable if not taking part in interviews)** |  |  |
| 6 | I know that the team might share results from the research in reports, conferences, etc. but this will not contain any names or identifiable information. |  |  |
| 7 | I know that the team might share information with other researchers in the future, but this will not contain any names or identifiable information. |  |  |
| 8 | I agree to / I agree for the young person I care for to take part in this research. |  |  |

**Please fill in your details below:**

Name of young person:

Signature: _______________________________________

Date: ___________________________________

Name of parent/guardian: _____________________________

Signature: __________________________________

Date: ___________________________________

If you have any questions or change your mind, you can talk to your teacher or contact the research team as follows:

**Tamanna Malhotra: txm390@bham.ac.uk**

**Alison Fulop: axf551@bham.ac.uk**

Kate Woodcock (study director): k.a.woodcock@bham.ac.uk

**Please see below for how to email your permission instead of filling in this form:**

**If you would prefer to send us an email instead of filling in the form, please send us a copy of the email below to Alison Fulop at** [**axf551@bham.ac.uk**](mailto:axf551@bham.ac.uk)

**If you are under 16, please ask your parent / legal guardian to send us a copy of the email below. Please ask them to write their name and your name in the places indicated in red.**

*My name is [insert parent / legal guardian name] I am the parent / legal guardian of [insert young person’s name]. We have read the Discussions about Lina information sheet and consent form. We would like to give our permission for [insert young person’s name] to take part.*

**14. YOUNG PERSON DEBRIEF LETTER**

**Debrief Letter**

**Discussions about *Lina***

**Study Director: Dr. Kate Anne Woodcock**

Thank you for your participation in *Discussions about Lina*.

Your participation will inform the development and wider-scale implementation of *Lina*. We will be using the collected data to better understand the ways in which *Lina* can be improved, and how it increases belongingness and wellbeing for young people in schools.

We will be keeping you informed about the findings of the study as and when they are published. We will also share any information on any further developments to *Lina*.

If you have any questions, or if you would like regular detailed updates at each step of the study, then please feel free to contact the research team as follows:

**Tamanna Malhotra: txm390@bham.ac.uk**

**Alison Fulop: axf551@bham.ac.uk**

Kate Woodcock (study director): k.a.woodcock@bham.ac.uk

Phone Number: 0121 414 6036

If anything you have talked about with us today has made you feel uncomfortable, please consider the information and resources below:

If you feel like you need some support with any negative feelings, please reach out to (insert bespoke school option).

Information regarding mental health and wellbeing resources is given below:

● If you're feeling overwhelmed and like you can't cope, you can:

(A) Call HOPELINEUK on 0800 068 4141 or the Samaritans on 116 123.

(B) Text SHOUT to Shout's textline on 85258.

(C) Call the NHS on 111 and select option 2.

● For a longer list of helplines and organisations for young people, please visit - https://www.mind.org.uk/for-young-people/how-to-get-help-and-support/useful-contact s/

**Appendix 2**

**Version Control Log (ASP-Belong)**

| **Trial Title** | Augmented Social Play (ASP): smartphone-enabled group psychotherapeutic interventions that boost adolescent mental health by supporting real-world connection and sense of belonging |
| --- | --- |
| **Chief Investigator:** | Prof. Kate Woodcock |
| **Sponsor:** | University of Birmingham |

| **Document Title** | **Version Number** | **Date** | **Date approved**  **(REC)** | **Current/**  **Superseded?** |
| --- | --- | --- | --- | --- |
|  |  |  |  |  |
|  |  |  |  |  |
| **Protocol** |  |  |  |  |
|  |  |  |  |  |
|  |  |  |  |  |
| **Consent/Assent Forms Standard Operating Protocols** |  |  |  |  |
|  |  |  |  |  |
|  |  |  |  |  |
| **Standard Operating Protocols** |  |  |  |  |
|  |  |  |  |  |
|  |  |  |  |  |
| **LOGs** |  |  |  |  |
|  |  |  |  |  |
|  |  |  |  |  |
| **Case Report Form (CRF)** |  |  |  |  |
|  |  |  |  |  |
|  |  |  |  |  |
| **Recruitment material** |  |  |  |  |
|  |  |  |  |  |
|  |  |  |  |  |
| **CRF** |  |  |  |  |
|  |  |  |  |  |
|  |  |  |  |  |

1.	Hards E, Loades ME, Higson-Sweeney N, Shafran R, Serafimova T, Brigden A, et al. Loneliness and mental health in children and adolescents with pre-existing mental health problems: A rapid systematic review. British Journal of Clinical Psychology. 2022;61(2):313-34.

2.	Gökmen Arslan PD. School belongingness, well-being, and mental health among adolescents: exploring the role of loneliness. Australian Journal of Psychology. 2021;73(1):70-80.

3.	Eugene DR. Connectedness to Family, School, and Neighborhood and Adolescents’ Internalizing Symptoms. International Journal of Environmental Research and Public Health. 2021;18(23).
